# Neural responses to state curiosity in young children

**DOI:** 10.1016/j.dcn.2026.101687

**Published:** 2026-01-28

**Authors:** Maayan S. Ziv, Monica E. Ellwood-Lowe, Morgan Botdorf, Monami Nishio, Elizabeth Bonawitz, Allyson P. Mackey

**Affiliations:** a Department of Psychology, School of Arts and Sciences, University of Pennsylvania, United States; b Applied Clinical Research Center, Children’s Hospital of Philadelphia, United States; c Graduate School of Education, Harvard University, United States; d Graduate School of Education, Stanford University, United States

**Keywords:** Curiosity, Learning, Memory, Neural activation, Children, FMRI

## Abstract

Curiosity scaffolds children’s exploration and learning. Yet, the neural mechanisms of curiosity-modulated learning in children remain unclear. Here, we designed an fMRI task to test how curiosity, as defined by children’s self-reported excitement about learning information, modulates memory and neural activity in 5- to 8-year-olds (*n* = 60 with behavioral data, *n* = 51 with fMRI). We observed greater learning when children reported more curiosity. In whole-brain analyses, high-curiosity was associated with greater activation in inferior frontal gyrus, lateral occipital cortex, the thalamus, and the putamen. Curiosity did not modulate activation in preregistered regions of interest (dorsal attention network, hippocampus, nucleus accumbens) but did modulate activation in an exploratory region of interest, the amygdala. Multivariate searchlight decoding revealed local activity patterns that reliably distinguished reported curiosity levels in dorsolateral prefrontal cortex, fusiform gyrus, angular gyrus, precuneus, and cerebellum. Together, these findings are consistent with prior work on curiosity-related activation during information receipt in adults, suggesting that neural systems that support curiosity-driven learning are already engaged in early childhood.

## Highlights

• We examined how state curiosity modulates memory and neural activation in children.
• Children learned more about items for which they reported greater curiosity.
• High curiosity engaged language, visual, and attention-related brain regions.
• We found distinct neural patterns for curiosity states in cognitive control regions.

## Graphical Abstract

**Figure:**
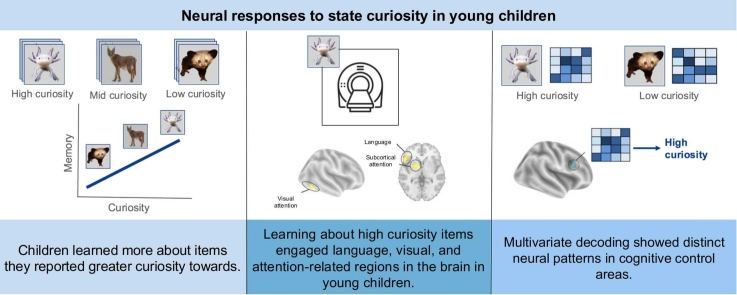

## 1 Introduction

In early childhood, curiosity may play a key role in guiding attention and supporting learning, but how this process unfolds in the brain remains unclear (for review, see Gruber and Fandakova, 2021). Aspects of trait curiosity, including information seeking and enjoyment of learning, are associated with a broad range of positive cognitive, social, and academic outcomes (Gottfried et al., 2023, Jovanovic and Brdaric, 2012, Lin et al., 2024, Mussel, 2013, Shah et al., 2018). Importantly, however, curiosity is not static within individuals; different stimuli and contexts elicit fluctuations in curiosity, as often characterized by exploratory play (Bonawitz et al., 2011, Yu et al., 2018), and transient states of heightened curiosity enhance learning and memory (Gruber et al., 2014, Jirout and Evans, 2023). The link between curiosity and learning behaviors raises questions of how curiosity and learning are linked at the mechanistic level. In particular, understanding the neural mechanisms of curiosity-modulated learning provides insight into the broader processes that support learning: examining how curiosity engages children’s attention, memory, and reward systems elucidates how these potential contributing factors support learning. This in turn may inform educational interventions by helping researchers design approaches that isolate and support those factors most relevant for learning. While considerable progress has been made in characterizing these mechanisms in adults, we do not yet know how curiosity supports learning in children (for review, see Gruber and Fandakova, 2021).

Theoretical and empirical work suggests that curiosity functions to enhance learning through both behavioral and cognitive-encoding factors. Behavioral factors suggest that curiosity leads to motivated information-seeking (Kidd and Hayden, 2015). Curiosity encompasses cognitive and affective processes that drive individuals to seek new information and resolve uncertainty (Jirout et al., 2024). According to the information-gap theory, curiosity arises when individuals perceive a gap between what they know and what they want to know, creating a cognitive tension that motivates information-seeking (Loewenstein, 1994). This theory is substantiated by findings that adults willingly exchange monetary rewards for answers to trivia questions they are curious about (Kang et al., 2009). Similarly, non-human primates exchange primary rewards for information. Macaques, for instance, will forgo water to gain non-instrumental information about future outcomes, demonstrating an intrinsic value of information to reduce uncertainty and improve predictions (Tian et al., 2021, Bromberg-Martin and Hikosaka, 2009). Curiosity may also enhance learning by preparing the mind for new information in a way that supports encoding (Begus and Bonawitz, 2020). Consistent with the theory that curiosity functions to support learning, adults demonstrate greater memory for trivia questions that elicit high curiosity states, and this effect extends to incidental information presented during high curiosity states as well (Kang et al., 2009, Gruber et al., 2014). Although curiosity-modulated learning has been widely substantiated in adults, relatively less is known about how state curiosity interacts with memory in children.

A few studies have investigated the impact of curiosity on memory in children. One study found that children aged 10- to 14-years-old showed better memory for trivia questions they rated as eliciting greater curiosity (Fandakova and Gruber, 2021). Another study observed a similar effect in 10- to 12-year-olds, though this replication relied on a relatively small sample of 32 children (van Schijndel and Jansen, 2025). Evidence in younger age groups is more limited, in part because state curiosity is difficult to operationalize and measure reliably in early childhood. One cross-sectional study found that 4- to 9-year-olds with greater interest in dinosaurs showed better recall of dinosaur facts after visiting a dinosaur museum exhibit (Festini et al., 2025). Thus far, one study has examined the relationship between state curiosity and memory in children under 10 (Jirout and Evans, 2023). This study operationalized state curiosity in 4- to 9-year-olds as the number of questions they asked and found that greater question-asking was associated with better memory for facts heard during an information-seeking task. However, since this study employed a between-child design, it is possible that children who asked more questions also had better memory. Thus, additional studies are needed to capture the impact of within-child fluctuations in state curiosity on learning.

To substantiate the link between curiosity and learning at the neural level, studies have employed neuroimaging in nonhuman primates and human adults to explore brain systems involved in curiosity modulation of learning. In nonhuman primates, distributed neural systems coordinate activity across reward, memory, and control regions to support curiosity-driven learning (Monosov, 2024). In adults, curiosity modulates memory by recruiting dopaminergic neural systems, enhancing hippocampal-dependent learning.

Studies investigating the neural basis of curiosity-modulated learning in adults distinguish between two complementary phases of curiosity: elicitation, when curiosity is triggered by uncertainty or information gaps, and satisfaction, when curiosity is resolved upon receipt of new information (Gruber et al., 2014, Kang et al., 2009, Ligneul et al., 2018). One study asked adult participants trivia questions and recorded neural activation during both the anticipation of trivia answers and the subsequent receipt of information. During anticipation, higher curiosity was associated with greater activation in dopaminergic midbrain regions, including the substantia nigra/ventral tegmental area and nucleus accumbens, and this anticipatory activity predicted later memory performance. In contrast, curiosity-related effects were not observed in dopaminergic regions during information receipt, suggesting that curiosity enhances learning through dopaminergic modulation of hippocampal encoding mechanisms during anticipation (Gruber et al., 2014).

Other studies have substantiated this distinction between neural responses during curiosity elicitation (or anticipation) and satisfaction (or receipt), showing that curiosity unfolds as a dynamic process engaging partially overlapping but temporally distinct neural systems (Duan et al., 2020, Ligneul et al., 2018, Van Lieshout et al., 2018). During information receipt, curiosity-related activation has been observed in the inferior frontal gyrus, parahippocampal gyrus, fusiform gyrus, putamen, insula**,** orbitofrontal cortex**,** and anterior cingulate cortex, regions implicated in processing information (Kang et al., 2009, Van Lieshout et al., 2018). One study observed curiosity-related activation during information receipt in the ventral striatum, with stronger activation when answer delivery was uncertain, reflecting intrinsic reward of gaining information (Ligneul et al., 2018). Furthermore, decisions to satisfy curiosity recruit the same dopaminergic reward circuits as extrinsic motivators like hunger, with both curiosity and hunger eliciting activation in the nucleus accumbens (Lau et al., 2020).

While functional neuroimaging studies have not yet explored neural responses to state curiosity in children, neuroscientific theories of curiosity suggest that the neural mechanisms supporting curiosity-modulated learning become more specialized with age. As the anterior cingulate cortex and lateral prefrontal cortex continue to mature, adolescents may be better able to regulate and sustain curiosity in ways that enhance memory, particularly when learning involves uncertainty or surprise (Gruber and Fandakova, 2021). However, some behavioral studies have suggested that curiosity may decline across development, indicating that neural mechanisms supporting curiosity may already be engaged in early childhood (Engelhard and Monsaas, 1988, Liquin and Gopnik, 2022). However, these hypotheses remain largely theoretical, and it is possible that curiosity-modulated learning may rely on distinct neural systems in children.

Other neuro-developmental work, framing curiosity as a system that is engaged when there is an expectation of information, has suggested that multiple regions of the brain are coordinated through phase synchrony when curiosity is heightened (Begus and Bonawitz, 2020). One argument is that curiosity supports learning via slow oscillation, such as theta activity, because it helps connect many neurons in large brain areas, and thus facilitates information transfer between different structures of the brain, including memory and reward centers. A recent study with 16-month-old infants showed a link between infants who tracked the expected informativeness of information, as measured by heightened theta response on high-expectation trials, and their causal learning; this provides an additional association between theta activation across diverse brain areas, curiosity, and learning in early childhood (Begus and Bonawitz, 2024). However, as a cortical EEG measure, this work could not measure the precise brain regions that were engaged during the task, and thus it remains unclear what specific neural systems were engaged.

Here, we examine how state curiosity relates to memory and neural activation during learning in young children. We modified a classic adult fMRI curiosity learning paradigm for typically developing children ages 5- to 8-years-old (Gruber et al., 2014). To align our learning stimuli with the United States’ Next Generation Science Standards (NGSS) for early elementary education, children learned about uncommon animals’ habitats and food (NGSS Lead States, 2013). Before scanning, we asked children to sort animals into high-, mid-, and low-curiosity categories by asking them how excited they were to learn about each animal. We collected fMRI data while the children learned about each animal’s habitat and food, and tested children’s recognition and recall following the scan. We explored whole-brain group average contrasts between learning during high and low curiosity states. In preregistered analyses, we tested neural activation in regions selected to represent three neurocognitive systems that have been theoretically related to children’s curiosity-driven learning: attention (dorsal attention network) memory (hippocampus), and motivation (nucleus accumbens) (Kidd and Hayden, 2015, Jirout et al., 2024). We hypothesized that high curiosity states would activate these regions in young children. In an exploratory analysis, we performed multivariate searchlight decoding to identify brain regions with activation patterns that reliably distinguish between high- and low-curiosity states.

## 2 Methods

This study was approved by the Institutional Review Board of the University of Pennsylvania. All parents provided informed, written consent. Children under the age of eight years provided verbal assent, and children over eight years provided written assent. Preregistered analyses were outlined in AsPredicted (#209157). The preregistration specified ROI analyses as focused tests of our hypotheses and noted that whole-brain contrasts (each curiosity level vs. rest and high vs. low curiosity) would be conducted as well. We preregistered a target sample of 50 participants.

### 2.1 Participants

Children in Kindergarten through 2nd grade (*ages: M* = 6.95, *SD* = 0.84) were recruited from the greater Philadelphia area as part of a larger study focusing on neural predictors of science learning. Recruitment occurred through social media advertisements and local community events. Parents completed screeners prior to participation and exclusion criteria were set to align with a larger study on neural correlates of science learning. Children were excluded if they were born more than six weeks premature, were adopted, had a diagnosed psychiatric, neurological, or learning disorder, or had any contraindications for MRI scanning. Sixty-one children participated; 60 yielded usable behavioral data (exclusions: fell asleep during learning task in scanner [n = 1]), and 51 yielded usable T1-weighted and fMRI data (exclusions: scanner malfunctioned [n = 2], mean framewise displacement > 1 mm [n = 7]) (see Fig. S1). Descriptive statistics of participants are shown in Table 1. The sample with usable fMRI data did not differ from the full sample in age, sex, or household income (see Table S1).

**Table 1:** Descriptive statistics of full participant sample (*n* = 61).

**Age**		*M =* 6.95 *(SD* = 0.84)		
		range = 5.25–8.79		
				
**Sex**				
Male		31 (50.8 %)		
				
**Race**				
White		45 (73.8 %)		
Black		12 (19.7 %)		
Asian		8 (13.1 %)		
Other		2 (3.3 %)		
				
**Ethnicity**				
Hispanic/Latino		9 (17.6 %)		
				
**Household income**		*M = $164,180 (SD* = $58,014)		
		range = $8,500-$200,000		
				

### 2.2 Experimental design

**Fig. 1:**
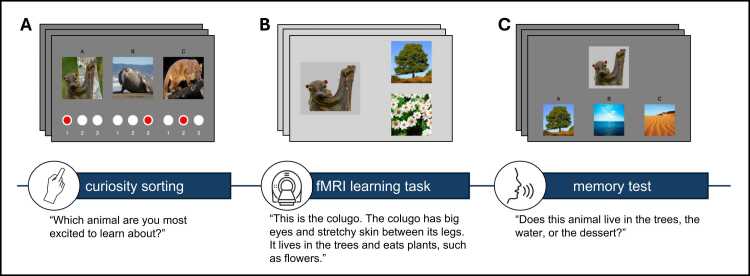
The curiosity learning task design. (A) Participants ranked curiosity about three animals for nine consecutive trials. (B) During scanning, participants learned about animals’ habitats and food across 27 trials. (C) After scanning, a memory test assessed recognition and recall of the animals’ habitats and food.

Participants completed a three-part paradigm that involved a curiosity sorting phase (Fig. 1A), a learning phase (Fig. 1B), and a recognition and recall test phase (Fig. 1C). Young children are not yet proficient readers, have limited ability to use response buttons in the scanner, and often struggle to interpret abstract rating scales like Likert measures (Mellor and Moore, 2014). To accommodate these constraints, we implemented a pre-scan curiosity-sorting phase in which children verbally selected which animals they were “most excited to learn about” (Fig. 1A). This approach allowed us to infer relative curiosity levels across stimuli in a child-friendly and interactive format. We obtained bin-level (high, medium, low) curiosity ratings. We did not ask children to rate animals across different sets to avoid repeating questions about previously seen stimuli, as young children often change their responses when asked repeated questions, interpreting repetition as an indication that their initial answer was incorrect or undesirable (Bonawitz et al., 2020). During scanning, children passively viewed and listened to 12-second narrated stories about the animals, ensuring standardized sensory input while reducing task demands (Fig. 1B). We measured brain responses during the receipt of information to maintain task feasibility and engagement in a young sample. The curiosity sorting phase occurred prior to scanning, the learning phase occurred during fMRI scanning, and the test phase was completed after the scan, approximately 15 min following the learning phase (Fig. 1C). Study tasks were created and implemented using PsychoPy (v2022.2.3), an open-source software for behavioral experiments (Peirce et al., 2019). The experimenter script used in the study protocol is included in Appendix S2.

This task was developed through extensive behavioral piloting. In pilot data collected at a children’s museum, we also examined the effect of extrinsic rewards on children’s attention and memory. A subset of animals was randomly outlined with green boxes, and children were told they would receive a prize for each green-boxed animal remembered, with the prize box shown to them beforehand. While children’s curiosity ratings predicted memory performance in the pilot sample (*n* = 24), we observed no effect of the extrinsic reward manipulation. These findings provided proof of concept for the task design, suggesting that children’s subjective curiosity ratings may meaningfully reflect motivational states relevant to learning. The reward component was excluded from the final experiment to reduce task complexity and maximize statistical power. Although our curiosity-sorting procedure provides lower resolution than trial-by-trial ratings, the pilot results support its validity as a meaningful measure of children’s intrinsic motivation, offering a developmentally appropriate approach to assessing curiosity in young participants. Pilot data is available on OSF.

#### 2.2.1 Curiosity-sorting phase

During scan preparation, research assistants introduced children to the "animal learning game." Children were told that in the first part of the game, they would see pictures of animals they probably had not encountered before and select which ones they were most excited to learn about. Children were presented with nine sets of three animals (three sets of mammals, three sets of birds, and three sets of reptiles or fish). Children were randomized to receive one of two versions, which differed in how animals were grouped into sets. The order in which sets were presented was randomized for all participants. Children began with a practice set featuring different dog breeds to familiarize them with the task. For each set, the experimenter asked children which animal they were most excited to learn about and assigned that animal a curiosity rating of 1 (high). The child then selected their next preferred animal in the set, which was assigned a curiosity rating of 2 (mid), while the remaining animal was given a rating of 3 (low). Thus, 27 animals were assigned curiosity ratings of high, mid, or low. Curiosity ratings assigned to each animal varied across children. All animals received high, mid, and low curiosity ratings, suggesting that children’s relative curiosity about animals differed (see Fig. S2). Additionally, we extracted visual properties from each animal image and found no relationship between children’s curiosity ratings and image properties, including brightness, intensity variance, global contrast, and color variance (see Fig. S3).

#### 2.2.2 Learning phase

The learning phase took place during fMRI scanning and was divided into two task runs, one with 14 animal stories and the other with 13 animal stories. The PsychoPy task was coded to randomize the order of animals presented within each run to minimize order effects. Children were shown pictures of each animal with corresponding images of the animal’s habitat and food, while listening to a simultaneous audio recording. The audio provided a brief story about the animal, including its name, where it lives, and what it eats. All animals lived in the water, trees, or desert, but foods were unique to each animal. Each habitat (water, trees, and desert) included animals from all three categories (mammals, birds, and reptiles) to prevent children from inferring habitats based on category membership. Approximately three seconds of rest was included in between each animal story with a jitter. Before the first run, children were told that they would be learning about the animals they had seen earlier and were instructed to pay attention, as they would be asked questions about the animals later. Functional MRI scans were collected throughout the learning task to capture neural activity associated with the task blocks.

#### 2.2.3 Recognition and recall phase

Following the scanning procedure, children completed the memory test phase with a research assistant, which included recognition and recall tasks. During the recognition task, children were shown 12 animals and tested on their recognition: 9 from the learning task (3 animals selected from each curiosity rating) and 3 lure animals not included in the learning task. The recall task consisted of two parts: habitat recall and food recall. The recall tasks used a three-alternative forced-choice paradigm to assess memory for learned facts about each animal. For each part, children were presented with 9 animals (3 from each curiosity rating) and asked to match each animal to its corresponding habitat or food. In the habitat recall task, participants were shown images of the three habitats used in the task (water, desert, and trees) and asked to select the corresponding habitat for each animal. In the food recall task, participants selected each animal’s corresponding food from three choices. Food lures were selected from foods corresponding to other animals in the task that belonged to the same food category (animal, plant, or bug). For example, the correct food for the verdin was caterpillars, and the lures were worms and grubs, both of which appeared in the task as correct foods for other animals (for the full experimenter protocol, see Appendix S2). We limited the post-scan memory task to a subset of stimuli from the learning phase to keep the task short and minimize fatigue. Children were randomized into one of three different orders that determined which sets of animals were selected for the recognition and recall trials.

### 2.3 Neuroimaging data acquisition

Imaging was performed at the Center for Advanced Magnetic Resonance Imaging and Spectroscopy (CAMRIS) at the University of Pennsylvania. Scans were conducted using a Siemens MAGNETOM Prisma 3-Tesla MRI scanner with a 32-channel head coil. A whole-brain, high-resolution, T1-weighted 3D-encoded multi-echo structural scan (MEMPRAGE) was collected (acquisition parameters: TR = 2530 ms, TI = 1330 ms, TEs = 1.69 ms/3.55 ms/5.41 ms/7.27 ms, BW = 650 Hz/px, 3x GRAPPA, flip angle = 7°, voxel size = 1 mm isotropic, matrix size = 256 × 256 ×176, FOV = 256 mm, total scan time = 4:38). This sequence used interleaved volumetric navigators to prospectively track and correct for subject head motion (Tisdall et al., 2012). Two T2 * -weighted gradient echo multiband EPI functional scans were also collected (acquisition parameters: multiband acceleration factor = 3, TR = 2000 ms, TE = 30.2 ms, BW =1860 Hz/px, flip angle = 90°, voxel size = 2 mm isotropic, matrix size = 96 × 96, 75 axial slices, FOV = 192 mm, volumes = 150, 5 dummy scans). Participants completed the animal learning task during functional scans, and PsychoPy (v2023.2.3) was used to ensure the beginning of each functional scan triggered the start of the task. PsychoPy was also used to record the onset of each trial, which was subsequently used for defining event blocks in task analysis. Children’s scanner motion across trials did not differ by curiosity (see Fig. S4).

### 2.4 Behavioral data analysis

Behavioral data were analyzed in R using linear mixed-effects models in the lme4 package to account for within-subject variability across trials (Bates et al., 2015). Accuracy was predicted by curiosity level (high, mid, low), with random intercepts for participant and age included as covariates. Separate models were estimated for each memory task (recognition, habitat recall, and food recall) to assess the effect of curiosity on learning performance across task types.

### 2.5 MRI data preprocessing

Synthetic BOLD Contrast for Distortion Correction was used to generate fieldmaps (Yu et al., 2023). Functional MRI data were preprocessed using fMRIprep (version 24.1.1; Esteban et al., 2019). The preprocessing pipeline included brain extraction, motion correction, and spatial normalization to MNI152 space. Slice timing correction was applied using the middle of the repetition time as the reference point. Confounds, including three translations, three rotations, and six anatomical CompCor components, were regressed out of the data to account for motion and physiological noise. A high-pass filter with a cutoff frequency of 0.01 Hz was applied to remove low-frequency signal drift. Task data and confound regressors were trimmed to match the duration of the task.

### 2.6 Whole brain analyses

Regressors for blocks during which participants learned about animals were defined by participants’ curiosity ratings of each animal (high, mid, or low). Regressors were convolved with the Glover canonical hemodynamic response function and included in a first-level general linear model using Nilearn’s FirstLevelModel. The model incorporated an autoregressive noise model of order 1 and a cosine basis function drift model. Contrast maps comparing each curiosity state (high, mid, and low) to rest were generated for each run and combined across runs using a fixed-effects model within participants. Group-level analyses were conducted using Nilearn’s SecondLevelModel, with a design matrix including age and mean framewise displacement (demeaned) as covariates. Whole-brain z-statistic maps for group-level contrasts were retained for visualization and thresholded using a cluster-defining threshold of z > 3.1 (p < 0.001) and cluster > 10 voxels, and correction for false positive rates was applied. We computed contrasts comparing each curiosity condition to rest as well as direct pairwise contrasts between curiosity levels to identify regions where neural activation scaled with curiosity.

### 2.7 Region of interest analyses

Preregistered regions-of-interest (ROI) analyses focused on the nucleus accumbens, hippocampus, and dorsal attention network. The nucleus accumbens and hippocampus were defined using the Harvard-Oxford atlas, and the dorsal attention network was defined using the Yeo 7 functional atlas (Yeo et al., 2011). Z-scored contrast maps for each curiosity state were used to extract activation values from these ROIs. Linear mixed-effects models were used to examine the main effects of curiosity state (dummy-coded: low = 0, mid = 1, high = 2) and age on ROI activation, as well as their interaction, while accounting for individual differences with a random intercept for participants. As an exploratory analysis, we examined whether amygdala activation varied by curiosity level. Bilateral amygdala was defined using the Harvard-Oxford atlas, and median BOLD signal during learning blocks of each curiosity level was extracted for each participant.

### 2.8 Searchlight analysis

We conducted an exploratory searchlight analysis to examine multivariate patterns of neural activity associated with curiosity. Multivariate pattern analysis can detect distributed representational differences associated with curiosity that may not show up as mean activation differences (Davis et al., 2014). For each participant, we first estimated first-level general linear models separately for two functional runs using beta-series modeling. One beta map was generated for each trial (each animal story block) based on subject-specific design matrices constructed from event timing files. We used Nilearn’s FirstLevelModel, which included an autoregressive noise model of order 1, a cosine drift model, and no spatial smoothing. Regressors modeled the onset of each learning block, labeled by the animal’s name and convolved with the Glover canonical hemodynamic response function. To account for physiological noise, additional nuisance regressors included three translations, three rotations, and the first six anatomical CompCor components. The resulting high and low curiosity trial-level beta maps (9 trials per curiosity level; 18 total) were concatenated into a 4D image and labeled based on trial-level curiosity ratings.

The classifier was trained to discriminate high- versus low-curiosity trials to identify whether curiosity states could be decoded from distributed spatial activation patterns. Searchlight decoding was performed independently within each spherical searchlight using a linear support vector classifier (SVC) with 3-fold stratified cross-validation, ensuring balanced representation of high- and low-curiosity trials within each fold. A spherical searchlight of 5 mm radius was used, and classifier accuracy scores were computed at each voxel within a subject-specific brain mask.

The resulting accuracy maps were centered by subtracting chance performance (0.5) and group-level effects were computed using Nilearn’s SecondLevelModel. A one-sample *t*-test assessed whether decoding accuracy was significantly above chance across participants. Group-level statistical maps were thresholded using a cluster-level false positive rate (FPR) correction with a voxel-wise threshold of z > 3.1 and a minimum cluster size of 10 voxels. Resulting maps show clusters that reliably distinguished between high- and low-curiosity trials across participants.

## 3 Results

### 3.1 Behavioral analyses

**Fig. 2:**
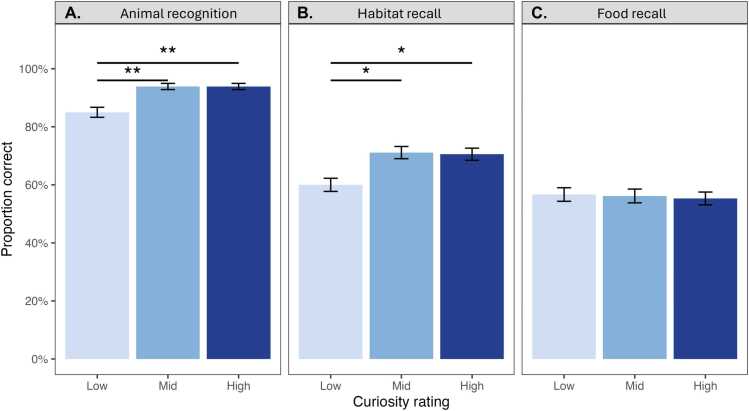
Learning outcomes by curiosity. Mean accuracy is displayed for each task type: A) recognition, B) habitat recall, and C) food recall. Error bars represent standard errors. Statistical significance was tested using a linear mixed-effects model with a random intercept for participant and age included as a covariate (*p < 0.05. **p < 0.01). Results indicate significant differences in accuracy across task types, with the highest accuracy observed for the recognition task, followed by habitat recall, and the lowest accuracy for food recall. Scores in all tasks were significantly above chance (50 % correct for recognition and 33 % for habitat and food recall).

As predicted, children learned more during trials rated as higher curiosity. Accuracy was significantly greater for higher curiosity trials, with effects observed for both recognition (β = 0.133, *SE* = 0.045, *p* = 0.004) and habitat recall (β = 0.158, *SE* = 0.073, *p* = 0.032), but not for food recall (β = −0.025, *SE* = 0.079, *p* = 0.755) (Fig. 2). For both recognition and habitat recall, high- and mid-curiosity trials showed significantly greater accuracy than low-curiosity trials (mid vs. low: β = 0.267, *SE* = 0.090, *p* = 0.004; high vs. low: β = 0.267, *SE* = 0.090, *p* = 0.004 for recognition; mid vs. low: β = 0.333, *SE* = 0.145, *p* = 0.023; high vs. low: β = 0.317, *SE* = 0.145, *p* = 0.031 for habitat recall), but we observed no difference between high- and mid-curiosity trials on animal recognition or habitat recall (β < 0.001, *SE* = 0.090, *p* > 0.999; β = 0.017, *SE* = 0.145, *p* = 0.909, respectively). The curiosity effect on recognition held when recognition performance was assessed using a sensitivity measure (d’) (Fig. S5).

Age predicted accuracy in the habitat recall task, with older children demonstrating higher performance (*β* = 0.197, *SE* = 0.088, *p* = .030). However, there was no significant interaction between curiosity and age on habitat recall accuracy (β = −0.099, SE = 0.088, p = 0.262). Age did not predict accuracy in either the animal recognition (*β* = −0.010, *SE* = 0.056, *p* = .861) nor food recall tasks (*β* = 0.114, *SE* = 0.093, *p* = .228) (see Fig. S6). Accuracy was highest for recognition and lowest for food recall (see Fig. S7). When data were combined across recall tasks, we observed main effects of both age and curiosity on memory performance (see Table S2). Curiosity and age effects remained robust when excluding participants who performed below chance on each task (see Table S3). All participants performed above chance when accuracy was aggregated across all task types (see Fig. S8 and Fig. S9).

### 3.2 fMRI analyses

#### 3.2.1 Whole brain analyses

**Fig. 3:**
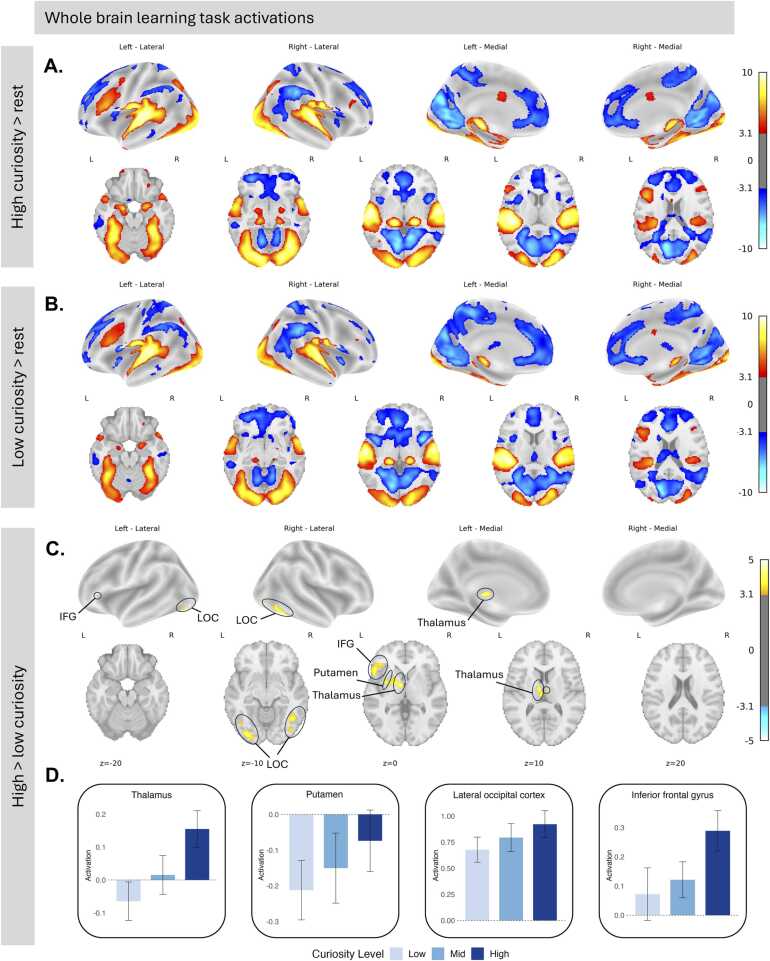
Neural activations during A) high- and B) low-curiosity learning stimuli compared to rest. Curiosity-related neural activations during C) high > low curiosity learning. Analyses control for age and motion (mean framewise displacement). Maps show z-statistics corrected for false positive rates with a cluster threshold of 10 and z > 3.1. D) Parameter estimates extracted from bilateral anatomical regions (Harvard-Oxford Cortical and Subcortical atlases) to demonstrate the pattern of activation compared to rest across curiosity levels. ROI data are shown for visualization purposes; no statistical tests were performed on extracted ROIs.

We performed whole-brain group analyses to examine task-related BOLD activation. First, we characterized overall task-related activation patterns. During high curiosity task blocks, we observed activation in visual (ventral stream) and auditory cortical and thalamic regions, as well as in dorsolateral prefrontal cortex (dlPFC). We also observed activation in the bilateral amygdala. The default mode network showed negative activation to the task during high curiosity task blocks, indicating greater activation of the default mode network during rest compared to task (Fig. 3A). A similar pattern of activation was observed during low curiosity task blocks (Fig. 3B). Activation maps for each curiosity level relative to rest, including the mid curiosity condition, are shown in Fig. S10.

Next, we directly contrasted high- versus low-curiosity trials to identify regions showing curiosity-related modulation of activity. Contrasts of high curiosity trials > low curiosity trials showed increased activation in visual processing regions, including the ventral visual stream, as well as subcortical attention regions, particularly the thalamus. Additionally, activation was observed in the left putamen (ventral), though this cluster did not clearly separate from the left thalamus. Increased activation during high curiosity states was also found in the left inferior frontal gyrus, a key region for language processing (Fig. 3C). These results remained robust in a sensitivity analysis excluding participants with mean framewise displacement > 0.5 mm on either task run (Fig. S11). Activation maps for the curiosity contrast are shown without statistical thresholding in Fig. S12, and MNI coordinates of significant clusters are shown in Table S4. Because whole-brain contrasts reflect relative differences between curiosity states, they do not indicate how activation during each condition compares to rest. To interpret these contrasts in relation to baseline activity, we extracted parameter estimates from anatomically defined regions (Harvard-Oxford Cortical and Subcortical atlases) to determine whether curiosity-related effects reflect increased activation above baseline or reduced suppression relative to rest (Fig. 3D) (Desikan et al., 2006). We also conducted a supplementary contrast comparing high + mid versus low curiosity trials, which showed a similar pattern to the high compared to low contrast, with greater neural activation in the fusiform gyrus and bilateral thalamic regions in high + mid compared to low curiosity (Fig. S13).

Finally, we examined whole-brain age effects. When we modeled a continuous effect of age, we saw greater activation in the ventral visual stream for older children during the task compared to rest (Fig. S14). However, we did not observe age-related differences in whole-brain activation patterns between high- and low-curiosity states.

#### 3.2.2 Preregistered region-of-interest analyses

We found no differences in nucleus accumbens, dorsal attention network, or hippocampal activation during learning by curiosity level. Neural activation in these regions did not vary by age, and there was no interaction between age and curiosity on activation in these regions (Table 2).

**Table 2:** Linear mixed effects model of main effect of curiosity, age, and their interaction on ROI activation to learning task.

	M1	M2	M3
A. Nucleus Accumbens			
Age	-0.005	-0.005	-0.037
	(0.096)	(0.096)	(0.110)
Curiosity	-	0.043	0.042
	-	(0.046)	(0.046)
Age x Curiosity	-	-	0.032
	-	-	(0.055)
Constant	-0.467	-0.510	-0.510
	(0.078)	(0.091)	(0.091)
B. Dorsal Attention Network			
Age	0.084	0.084	0.057
	(0.049)	(0.049)	(0.060)
Curiosity	-	0.025	0.025
	-	(0.029)	(0.029)
Age x Curiosity	-	-	0.027
	-	-	(0.035)
Constant	-0.120	-0.145	-0.145
	(0.040)	(0.050)	(0.049)
C. Hippocampus			
Age	0.083	0.083	0.094
	(0.075)	(0.075)	(0.086)
Curiosity	-	0.054	0.054
	-	(0.035)	(0.035)
Age x Curiosity	-	-	-0.011
	-	-	(0.042)
Constant	0.091	0.037	0.037
	(0.062)	(0.071)	(0.071)

*Note:* Mixed-effects models predicting (A) nucleus accumbens, (B) dorsal attention network, and (C) hippocampus activation to task. Model 1 (M1) includes age as a predictor. Model 2 (M2) includes age and curiosity as predictors. Model 3 (M3) shows the interaction between age and curiosity. All models include a random intercept for subject and control for motion (mean framewise displacement). Standard errors are shown in parentheses.

#### 3.2.3 Exploratory region-of-interest analysis: amygdala

**Fig. 4:**
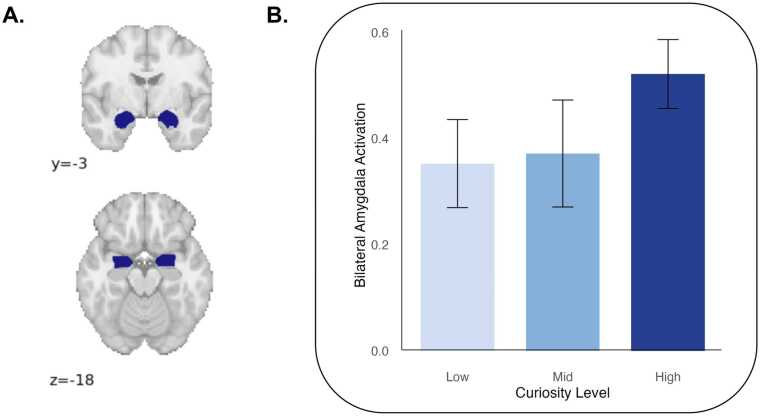
A) Bilateral amygdala B) activation during learning by curiosity level. Activation values show z-statistics. Group average activation is plotted across curiosity levels, and significance was tested using linear mixed effects models with a random intercept for participants and controlling for age and mean framewise displacement (β = 0.084, *SE* = 0.041, *p* = 0.043).

Given the strong bilateral amygdala activation for learning > rest in whole-brain contrasts, we explored amygdala activation to learning stimuli by curiosity level. Parameter estimates were extracted for each curiosity level (high, mid, low) relative to rest and entered into a linear mixed-effects model testing for a linear effect of curiosity. Curiosity predicted bilateral amygdala activation to learning stimuli (β = 0.084, *SE* = 0.041, *p* = 0.043) (Fig. 4).

### 3.3 Exploratory: searchlight decoding

**Fig. 5:**
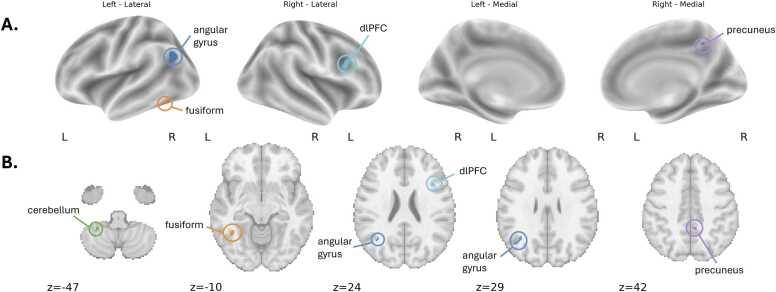
Regions with multivariate patterns related to curiosity level, as identified by searchlight decoding. **A)** Significant clusters (z > 3.1, cluster > 10 voxels, FPR-corrected) projected onto lateral and medial surfaces and **B)** shown on axial slices.

Searchlight decoding analyses were performed to localized patterns of neural activation that reliably distinguished between learning about high- and low-curiosity items. Curiosity-related neural patterns were localized in clusters spanning right dlPFC, left angular gyrus, left fusiform gyrus, right precuneus, and left cerebellum. Classifier accuracy in these regions significantly exceeded chance across participants (z > 3.1, cluster size > 10 voxels, FPR-corrected), indicating reliable discrimination of curiosity level from multivariate activation patterns. Parameter estimates extracted from each cluster revealed that the left fusiform gyrus and right dlPFC clusters activated above baseline during animal stories across curiosity levels (Fig. 5A-B). Clusters in left angular gyrus, right precuneus, and left cerebellum were suppressed below baseline across curiosity levels. Furthermore, univariate parameter estimates extracted from these clusters did not reveal significant group-level differences in average activation between high- and low-curiosity trials, indicating that the classifier’s performance likely reflects distributed multivariate patterns rather than localized differences in overall activation magnitude (Fig. S15).

## 4 Discussion

We examined how children’s reported state curiosity for different items predicted learning outcomes for those items and neural activation during learning. Children showed greater learning for items they reported more excitement to learn about. In whole-brain analyses, items with greater curiosity ratings were associated with greater activation in language areas (inferior frontal gyrus), visual areas (lateral occipital cortex), and subcortical regions including the thalamus and putamen. Additionally, amygdala activation was higher for items that children rated as higher curiosity. Multivariate searchlight decoding analyses showed that curiosity-related neural patterns were distinct in dlPFC, fusiform gyrus, angular gyrus, precuneus, and cerebellum. Together, these findings suggest that curiosity supports learning in young children by recruiting neural systems involved in language processing, visual attention, and attentional control.

Children showed greater learning for items they reported more excitement to learn about. The effect of curiosity on learning did not differ by age. Our findings that moment-to-moment fluctuations in curiosity predict learning in elementary school-aged children are consistent with prior studies demonstrating that heightened curiosity enhances memory in older children (Fandakova and Gruber, 2021, van Schijndel and Jansen, 2025). Curiosity effects were evident for recognition and for habitat recall, but not for food recall. This pattern may shed light on how curiosity interacts with the structure of memory tasks. Food recall required children to discriminate between highly similar alternatives, as lures were perceptually related foods seen elsewhere in the task. Habitat recall, in contrast, involved less perceptually similar options. Developmental work on episodic memory suggests that when representational similarity is high, children’s ability to engage in mnemonic discrimination and relational binding is taxed, leading to more errors (Ngo et al., 2019). Thus, curiosity may enhance memory strength broadly, but may not overcome difficulties in discriminating between overlapping representations. However, we did not expect task-specific effects, so future work is necessary to examine how curiosity interacts with mnemonic discrimination and relational binding demands in childhood. Additionally, recognition accuracy for mid- and high-curiosity items approached ceiling, potentially limiting our ability to detect a graded, linear relationship between curiosity and memory performance in that task. Our results extend the effect of state curiosity on memory to a younger age group, suggesting that the link between state curiosity and memory is already robust in early childhood.

Our findings are consistent with prior studies in adults showing curiosity-related neural activation during information receipt. We observed greater activation in inferior frontal gyrus during learning about high-curiosity items, consistent with prior work showing that high-curiosity states recruit inferior frontal regions during the receipt of new information (Kang et al., 2009). Similarly, activation in the putamen and thalamus has been observed during curiosity satisfaction, as the putamen is thought to encode the reward value of resolving uncertainty and the thalamus coordinates attentional gating through dopaminergic and thalamocortical pathways (Kang et al., 2009, Monosov, 2024). Enhanced activation in fusiform gyrus during high-curiosity learning has also been observed in adults, reflecting heightened perceptual processing that supports memory encoding (Kang et al., 2009). Finally, dlPFC has been implicated in the top-down control of curiosity-driven attention (Ligneul et al., 2018, Monosov, 2024). Together, these converging findings suggest that curiosity supports learning in young children by engaging distributed attentional, motivational, and perceptual systems.

We observed greater bilateral amygdala activation when children learned about high-curiosity items. Although the amygdala is canonically associated with fear states and has been hypothesized to suppress curiosity-driven exploration, the amygdala is broadly implicated in appraisal processes, and studies have shown that positive affective cues recruit the amygdala when stimuli relate to goal-directed learning (Gruber and Ranganath, 2019, Sander et al., 2003, Stillman et al., 2015). Moreover, the amygdala has been shown to activate during positive affective states in young children (Park et al., 2022). Theoretical accounts suggest that the amygdala is involved in appraising uncertainty, influencing whether a knowledge gap gives rise to curiosity-driven exploration or anxiety-driven avoidance (Erdemli et al., 2024). Thus, future work could investigate whether affective appraisal systems, including the amygdala, contribute to curiosity-modulated learning in children.

We did not observe curiosity-related activation in the dorsal attention network, which may be explained by the nature of the task. Because the task involved auditory and semantic processing, it may have recruited attention through engaging the ventral attention network. Additionally, the hippocampus was not significantly modulated by curiosity, consistent with prior findings in adults showing that curiosity states did not show a main effect on hippocampal activation in anticipation of or during learning (Gruber et al., 2014). While the prior study in adults observed a curiosity x memory interaction such that hippocampal activation predicted memory under high-curiosity conditions, we were underpowered to test this interaction in the current study (Gruber et al., 2014). We also did not find curiosity-related modulation of dopaminergic regions thought to underlie reward processing in children. This may be partly due to task demands: whereas prior studies have often examined curiosity-related neural responses during the anticipation of information, our design focused on brain activity during information receipt (Gruber et al., 2014). It may be that anticipation and receipt of information engage distinct neural systems, similar to adults.

Consistent with the univariate results, the searchlight MVPA revealed that the fusiform gyrus contained reliable spatial patterns distinguishing curiosity levels, recapitulating the univariate effects. Interestingly, we also identified distinct curiosity-related patterns in the dlPFC, a candidate region that may be a source of the control signal that modulates attentional engagement during curiosity. The dlPFC has been implicated in generating top-down control signals that bias attention toward task-relevant information, modulating posterior perceptual regions during learning and memory encoding (Ranganath et al., 2004). The identification of this region in the MVPA but not in the univariate analysis suggests that curiosity-related control signals may be encoded through changes in the spatial organization of activity rather than overall signal amplitude. In addition, three other regions showed distinct neural patterns for learning during high versus low curiosity items: the cerebellum, precuneus, and angular gyrus. Future work should further investigate these regions as potential contributors to curiosity-related control and learning processes.

A wide range of paradigms have been used to study curiosity in adults, including trivia tasks (e.g., Gruber et al., 2014), perceptual curiosity tasks with blurry-to-clear image transitions (e.g., Cohanpour et al., 2024; Jepma et al., 2012), and paradigms using magic tricks to elicit curiosity under uncertainty (Lau et al., 2020). These approaches share the goal of eliciting and measuring curiosity about forthcoming information and then measuring associated neural or behavioral responses. We designed our task to prioritize feasibility for young children in the scanner. Because some children struggle with continuous scales due to developing numeracy skills, we asked children to make simple decisions about their curiosity levels. Our fMRI design used naturalistic, narrated stories about animals to maintain engagement and minimize motion in young participants. Our task measured brain responses during learning but not during anticipation. To explore the boundaries between these contexts, future research could investigate the distinction between neural activation to curiosity elicitation and satisfaction in young children.

Several limitations of this study should be noted. First, to standardize the task at the group level, we relied on each child providing curiosity ratings for the same set of animals, assuming that children would vary in their curiosity across different stimuli. Furthermore, to allow for fMRI analyses with sufficient trials per condition, we needed to ask children to rank relative curiosity, limiting sensitivity to children’s absolute curiosity levels. It is likely that some children showed little differentiation in their curiosity, either showing low interest in all animals or high interest in all animals. While group-level results indicate that children, on average, learned more when they reported higher curiosity, individual variation in relative curiosity across trials may have introduced noise, potentially weakening the observed effects. Additionally, we measured neural activation during information receipt and did not have an anticipation phase in our design. Thus, we may have missed patterns of neural activation associated with the anticipation phase of learning about items that elicit high curiosity. Finally, we did not test correlations between neural activation during learning and subsequent memory performance. Because each memory task used a subset of the items learned, we had insufficient trials to examine brain-behavior relationships. Future work should explore how curiosity-related neural activity predicts memory in children.

Our results have important implications for educational practice. Educational interventions aimed at improving attention and engagement in classroom settings often focus on training children to filter sensory input and reduce distractions by maintaining eye gaze and body orientation as proxies for cognitive engagement (Lai and Chang, 2020, Psotta et al., 2023). The underlying assumption is that controlling where children look and what they listen to will enhance their processing of instructional material. However, our findings suggest that filtering children's sensory input may not be sufficient to recruit the visual and language processing systems that support learning and memory. Rather, eliciting children’s curiosity engages neural systems that maximize children’s learning. Our neuroimaging findings provide mechanistic evidence that curiosity enhances learning by modulating neural systems involved in attention and information processing. In sum, we observed that children learned more and showed greater neural activation in visual, language, and attention-related regions for high-curiosity items, and children’s self-reported curiosity was associated with distinct neural activity patterns in cognitive control regions.

## CRediT authorship contribution statement

**Monami Nishio:** Writing – review & editing. **Ziv Maayan S:** Writing – original draft, Visualization, Project administration, Formal analysis. **Ellwood-Lowe Monica E:** Writing – review & editing, Formal analysis. **Morgan Botdorf:** Data curation, Conceptualization. **Elizabeth Bonawitz:** Writing – review & editing. **Mackey Allyson P:** Writing – review & editing, Supervision, Project administration, Funding acquisition, Data curation, Conceptualization.

## Declaration of Competing Interest

The authors have nothing to declare.

## Data Availability

Raw data are available on OpenNeuro: https://openneuro.org/datasets/ds007185/versions/1.0.0. Code is available on Github: https://github.com/maayanziv27/2026_DCN_Curiosity_fMRI.

## Footnotes

Appendix A Supplementary data associated with this article can be found in the online version at doi:10.1016/j.dcn.2026.101687.
