# Supplementary material for: Neural responses to state curiosity in young children
Source: Dev Cogn Neurosci. 2026 Jan 28;78:101687. doi: 10.1016/j.dcn.2026.101687 (PMC12882712; doi:10.1016/j.dcn.2026.101687)
Supplement: Supplementary file 1 — Supplementary material [file mmc1.docx]

**Supplemental Materials: Neural Responses to State Curiosity in Young Children**

**Appendix S1.** Preregistration

**Figure S1.** Participants’ mean framewise displacement (mm) for each task run

**Table S1.** Descriptive statistics of participant demographics

**Appendix S2.** Experimenter protocol used in task administration

**Figure S2.** Proportion of high-, mid-, and low-curiosity ratings for animal stimuli

**Figure S3.** Mean curiosity ratings by perceptual properties of animal images

**Figures S4.** Children’s mean framewise displacement as a function of curiosity

**Figure S5.** Mean recognition sensitivity (d’) for high-, mid-, and low-curiosity items

**Figure S6. Learning by age for different curiosity levels across task types**

**Figure S7. Curiosity effects on memory performance across tasks**

**Table S2.** Behavioral mixed-effects model testing age, curiosity, task on memory performance

**Table S3**. Behavioral results excluding participants who performed below chance on each task

**Figure S8.** Participants’ accuracy across the recognition, habitat, and food recall tasks, shown as proportions correct

**Figure S9. Participants’ accuracy aggregated across recognition, habitat recall, and food recall tasks**

**Figure S10. Neural activations during high-, mid-, and low-curiosity learning blocks compared to rest**

**Figure S11.** Sensitivity analyses showing neural activations during high- and low-curiosity learning stimuli compared to rest and high > low curiosity learning stimuli

**Figure S12. Neural activations during high- and low-curiosity learning** stimuli **compared to rest and curiosity-related** neural activations **(without statistical thresholding)**

**Table S4.** MNI coordinates of significant clusters in high > low curiosity contrasts

**Figure S13.** Neural activations during high- and mid- curiosity learning stimuli versus low-curiosity learning stimuli

**Figure S14.** Age effects of neural activation during high-, mid-, and low-curiosity learning stimuli compared to rest

**Figure S15.** Parameter estimates of univariate activation for each cluster identified by searchlight decoding

**Supplemental References**

**Appendix S1. Preregistration**

***Neural responses to curiosity states in young children***(AsPredicted #209157)

**1) Have any data been collected for this study already?**
It's complicated. We have already collected some data but explain in Question 8 why readers may consider this a valid pre-registration nevertheless.

**2) What's the main question being asked or hypothesis being tested in this study?**
How do curiosity states modulate activation of the mesocorticolimbic circuit in young children? How does age relate to curiosity-driven modulation of mesocorticolimbic activation in early elementary school? We predict that children will show greater activation in nucleus accumbens, hippocampus, and the dorsal attention network in high versus low curiosity states. We hypothesize that younger children will show greater effects of curiosity state on activation in these regions.

**3) Describe the key dependent variable(s) specifying how they will be measured.**
Neural activation to high, mid, and low curiosity states will be measured in three regions of interest identified based on previous work showing their role in different processes in the dopaminergic modulation of learning: NAcc (reward processing), dorsal attention network (attention), and hippocampus (memory).

High, mid, and low curiosity states will be determined by children's curiosity ratings of novel animals prior to completing an fMRI learning task. High, mid, and low curiosity states will be identified as fMRI task blocks in which children learn about animals they give a curiosity rating of one, two, or three, respectively.

**4) How many and which conditions will participants be assigned to?**
None.

**5) Specify exactly which analyses you will conduct to examine the main question/hypothesis.**
Functional scans will be preprocessed using fMRIprep (version 24.1.1). Task-based fMRI data from each participant will be analyzed using Nilearn's FirstLevelModel. Event-related regressors for curiosity ratings (1, 2, or 3) will be convolved with the Glover canonical hemodynamic response function. The model parameters will be set as follows: a repetition time of 2, an autoregressive noise model of order 1 (ar1), and a drift model using cosine basis functions to account for low-frequency signal variations, a high-pass filter with a cutoff frequency of 0.01 Hz, the slice timing reference set to the midpoint of the repetition time, and a brain mask derived from the MNI152 atlas to restrict the analysis to brain regions. The design matrix will include the following confounds derived from preprocessing outputs: framewise displacement, rotations (X, Y, Z), translations (X, Y, Z), and 6 anatomic CompCor parameters. The scans and confound regressors will each be trimmed to match the end time of the task. Contrast maps will be generated to compare each curiosity state (1, 2, and 3) to rest, and contrast maps will be combined across each participant's task runs using a fixed effects model with Nilearn's SecondLevelModel.

Participants' z-scored contrast maps will be used to extract high, mid, and low curiosity state activation values from three regions of interest: nucleus accumbens (NAcc), hippocampus, and the dorsal attention functional network (DAN). The NAcc and hippocampus will be defined using the Harvard-Oxford atlas, and the DAN will be defined using the Yeo 7 functional atlas.

We will use linear mixed-effects models to examine the main effects of curiosity states and age on ROI activation, as well as their interaction, while accounting for individual differences by including a random intercept for participants. Curiosity states will be dummy coded such that low curiosity = 0, mid curiosity = 1, and high curiosity = 2. FDR correction will be applied to correct for multiple ROI comparisons.

Additionally, whole-brain activation maps will be retained to generate group-level contrasts of each curiosity state versus rest as well as high versus low curiosity states. Group-level analyses will be conducted using Nilearn's SecondLevelModel and the design matrix will include age and mean framewise displacement across both task runs (demeaned).

**6) Describe exactly how outliers will be defined and handled, and your precise rule(s) for excluding observations.**
T1w images will be rated for quality by two independent raters on a scale from 1 (perfect) to 4 (unusable). We will exclude subjects from analyses if their T1w image receives an average quality score greater than 3. Participants will also be excluded if their mean framewise displacement exceeds 1 mm in either task run, if raw images show artifacts or incidental findings, or if they fall asleep during the learning task. We will include participants regardless of behavioral performance on the post-scanner task because we are interested in individual differences in attention and memory.

**7) How many observations will be collected or what will determine sample size?
No need to justify decision, but be precise about exactly how the number will be determined.**
We will collect data until we have usable structural and fMRI data on 50 participants.

**8) Anything else you would like to pre-register?
(e.g., secondary analyses, variables collected for exploratory purposes, unusual analyses planned?)**
Data collection for this study is ongoing. In the existing data, fMRI images have been preprocessed. Individual and group level contrasts have been created to check that neural activation in learning blocks as compared to rest follows expected patterns. We have not looked at regions of interest or individual differences.

In an additional exploratory analysis, we will run linear models predicting the difference between high and low curiosity state activation in each ROI (separately) with exposure to formal schooling and age. Exposure to formal schooling will be measured as the total number of months a child has attended school (estimating the school year as September 1 – June 15). We will exclude participants who have been homeschooled from these analyses.


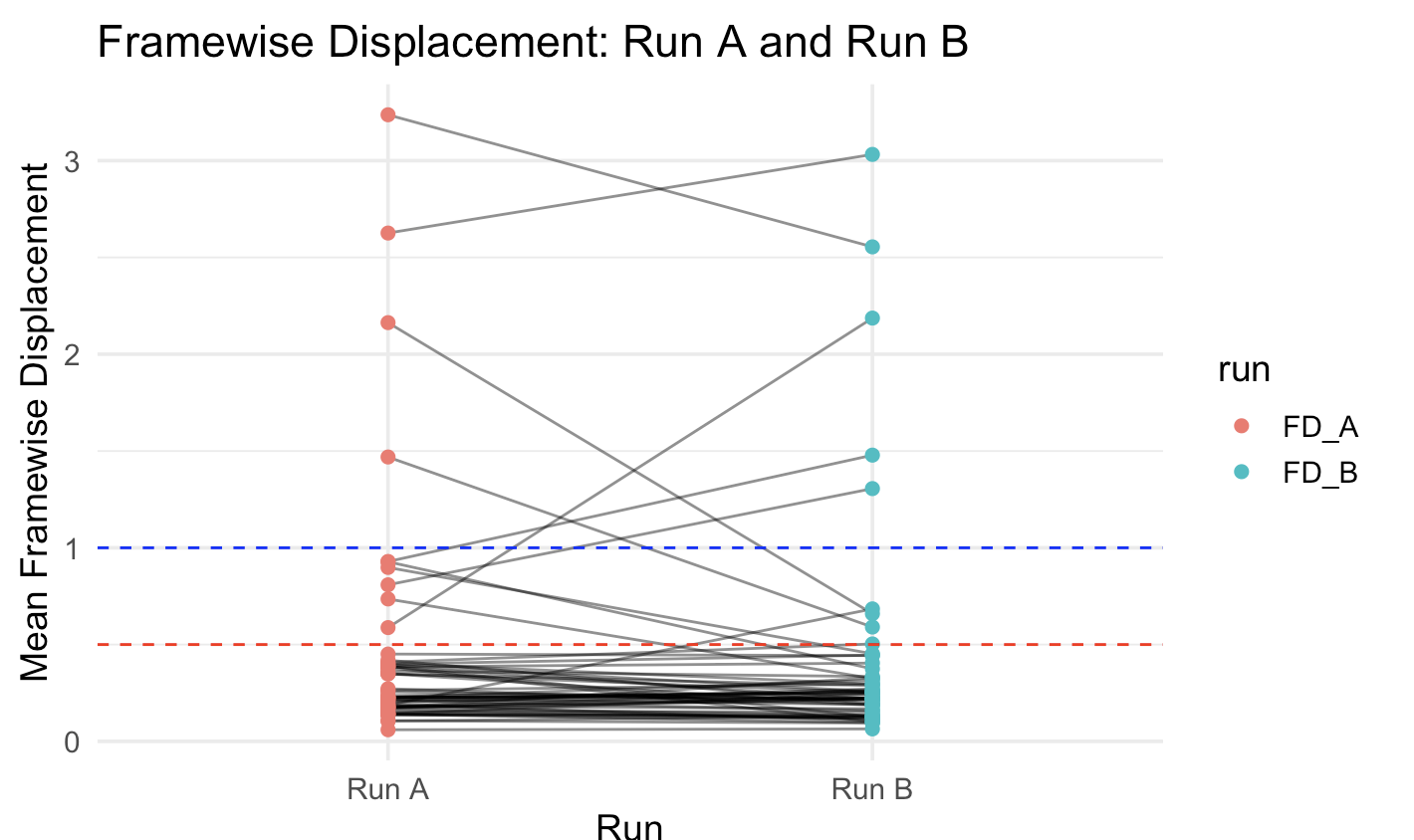


**Figure S1.** Participants’ mean framewise displacement (mm) for each task run. The blue and red dashed lines indicate 1 mm and 0.5 mm thresholds, respectively.

**Appendix S2.** **Experimenter protocol used in task administration**

Curiosity sorting phase experimenter script

1. Pre-task introduction
   1. “In this game, you’re going to learn about animals you probably haven’t heard of before. There is going to be 3 parts to this game. In the first part, you’ll see pictures of animals and you’ll tell me which ones you are excited to learn about. Then, in the 2nd part, you will hear stories about each animal. Finally, in the 3rd part, you’ll answer some questions about the animals you learned about in part 2.” “Do you have any questions about that?” “Let’s get started!”
2. Curiosity sorting phase
   1. General note: Give child praise as you're going through trials e.g., You're doing a great job paying attention! Just a few more left!
   2. “For this 1st part of the game, I’m going to show you pictures of animals and ask you if you are excited to learn about the animal. I want you to tell me first which one you’re most excited to learn about and I’ll mark a 1 for that one. Then tell me the next one that you’re most excited to learn about and I’ll mark a 2 for that one. Finally, the one that’s left will get a 3. I’m also going to write down your responses on my sheet of paper here so I can remember what you said. There are no right or wrong answers. Just tell me how you feel. Are you ready to get started?”
   3. “Great, give me one minute to open the game.”
      1. Pull up the curiosity sorting Psychopy file that you opened earlier. Click the green Run Experiment button (looks like a play button) located in the middle of the top toolbar. A screen will open that will ask you to input the subject number. Input the appropriate Participant ID and press Ok. Leave the Session ID as 001.
   4. Practice trial (1 trial)
      1. A gray screen will appear. Press the spacebar to move to the practice trial.
      2. “First, we’re going to do one practice round and then we’ll play for real. I have 3 animals on the screen here. Can you tell me which one you are most excited to learn about? You can point to the animal you want me mark as the one that you are most excited to learn about.”
         1. Experimenter clicks the 1 box that corresponds with animal the child is most excited about. A red circle will appear.
      3. “Of the two animals that are left, which one are you most excited to learn about?”
         1. Experimenter clicks the 2 box that corresponds with animal the child is most excited about. A red circle will appear.
      4. “I’m going to give the last animal that is left a 3”
         1. Experimenter marks the 3 in box that corresponds with animal the child is most excited about. A red circle will appear.
      5. At the end of the trial, ask the child: “Does that look right to you?”
         1. If child says yes, move onto the experimental trials
         2. If they say no, give them the opportunity to correct their answers
      6. “Great job! Now we’ll play for real.”
         1. After doing the practice trial, move on to the 9 experimental trials by pressing the spacebar.
         2. After each trial, remember to mark down the curiosity rating on the score sheet.
   5. Experimental Trials (9 trials)
      1. “I have 3 animals on the screen here. Can you tell me which one you are most excited to learn about? You can point to the animal you want me mark as the one that you are most excited to learn about.”
         1. Experimenter clicks the 1 box that corresponds with animal the child is most excited about. A red circle will appear.
      2. “Of the two animals that are left, which one are you most excited to learn about?”
         1. Experimenter clicks the 2 box that corresponds with animal the child is most excited about. A red circle will appear.
      3. “I’m going to give the last animal that is left a 3”
         1. Experimenter marks the 3 in box that corresponds with animal the child is most excited about. A red circle will appear.
      4. At the end of each trial, ask the child: “Does that look right to you?”
         1. If child says yes, record the curiosity ratings on the score sheet and then move on to next trial by pressing the space bar.
         2. If they say no, give them the opportunity to correct their answers
      5. After the task ends: “You’re all done with that part of the game!”

Scanning phase experimenter script

1. Before run A experimenter says:
   1. “*Now we are going to pause the movie, and you watch a new video on the animals you saw earlier with [research assistant]. You will learn what the animal looks like and see a picture of it. You will also learn where it lives, which is either in the water, in the trees, or in the desert and what it eats. You’ll see a picture of where it lives and what it eats below the animal. Be sure you pay attention while you learn about the animals because I will ask you questions about them later. While you’re learning about the animals, make sure you keep your arms, body, and most importantly head still like a statue!*”
2. Before run B experimenter says:
   1. “*You did a great job staying still! Can you give [research assistant] a thumbs up if you’re ready to watch the next part?*”

Post-test phase experimenter script

1. Introduction
   1. Say: *“Now, I am going to ask you some questions about the animals you learned about today during the animal learning game. Some of these questions may be easy and some may be hard. Just do your best to answer them. Do you have any questions?”*
2. Recognition Phase
   1. While the gray screen is up, say:
      1. *“In this first part, I’m going to show you a picture of an animal and ask you if you saw it today. You can say yes or no.”*
3. Press the spacebar to begin the recognition phase.
   1. For each animal shown:
      1. Ask: *“Did you see this animal today?”*
      2. Record response: yes = 1; no = 0.
      3. After all recognition trials, press the spacebar to move on to the recall phase.
4. Habitat Phase
   1. Say: *“For this next part, I’m going to show you an animal and ask you if you remember where the animal lives. You’ll see 3 pictures and you can tell me if it’s the 1st picture, 2nd picture, or 3rd picture.”*
   2. Press the spacebar to begin.
   3. Ask: *“Does this animal live in the [HABITAT 1], in the [HABITAT 2], or in the [HABITAT 3]?”*
      (Point to each picture as you name the habitat: trees, desert, or water.)
   4. Record response:
      1. Left picture = 1
      2. Center picture = 2
      3. Right picture = 3
5. Food Phase
   1. Say:*“Next, I’ll show you an animal and ask you if you remember what it eats. Again, you can tell me if it’s the 1st picture, 2nd picture, or 3rd picture.”*
   2. Press the spacebar to begin.
   3. Ask:*“Does this animal eat [FOOD 1], [FOOD 2], or [FOOD 3]?”*
      (Point to each picture as you name the food).
   4. Record response:
      1. Left picture = 1
      2. Center picture = 2
      3. Right picture = 3
6. Say: *“We are all done with our game! You did a great job!”*

**
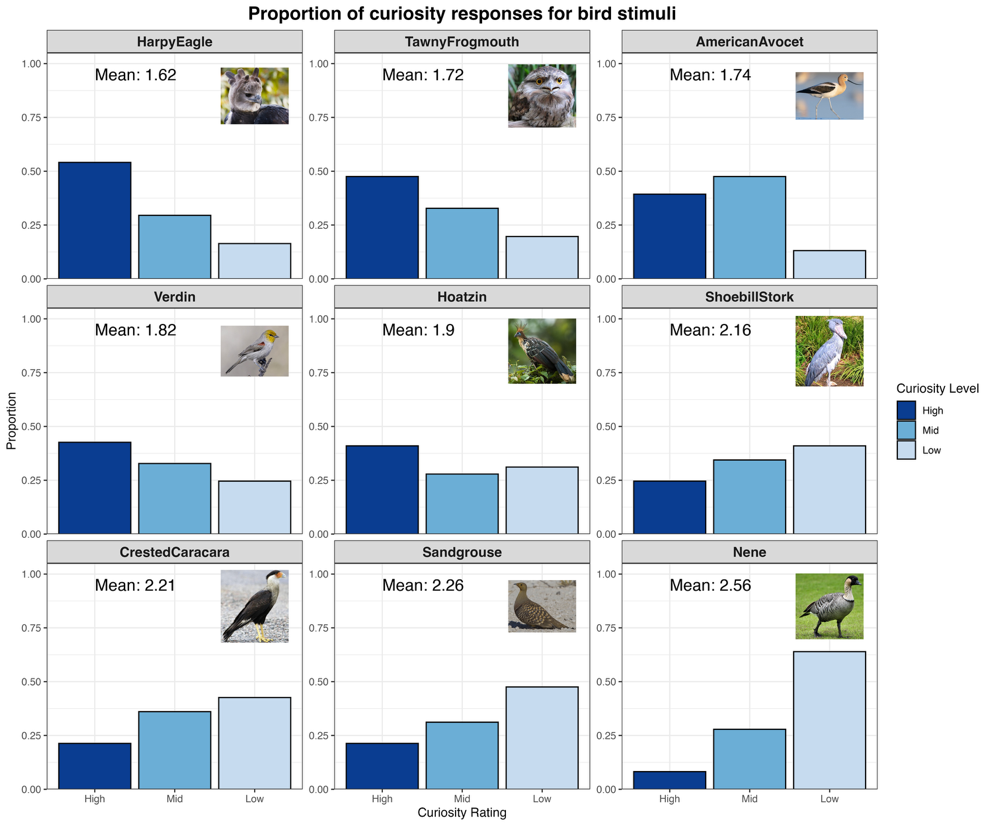
**
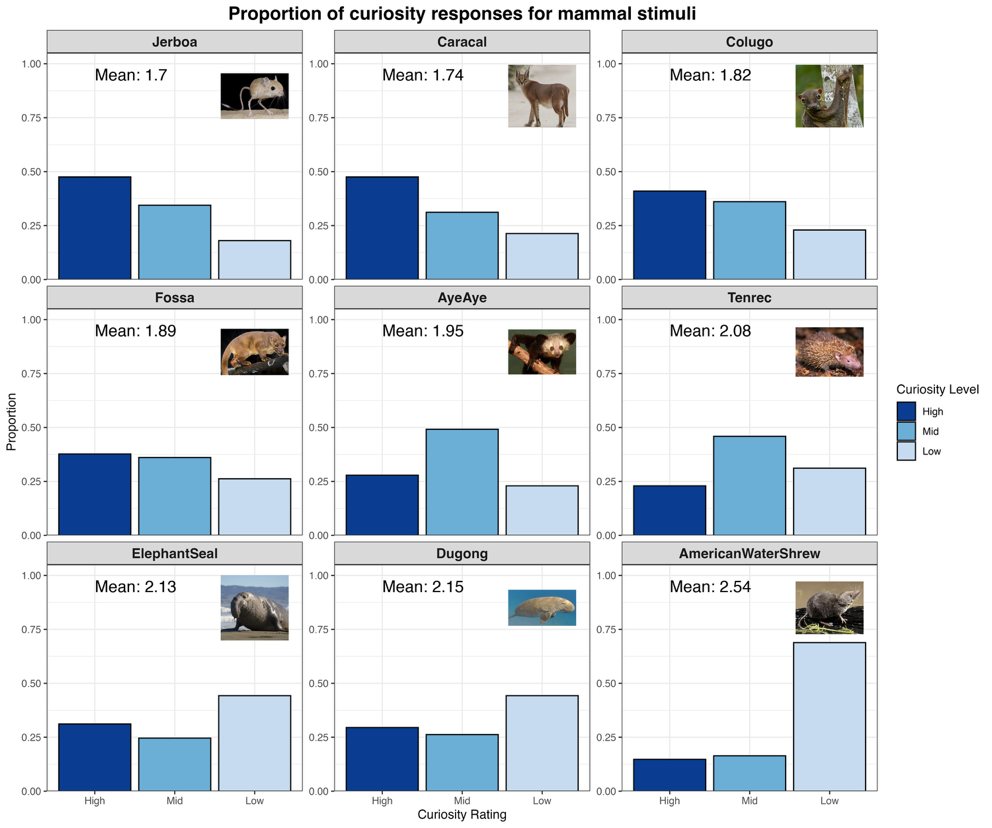


B.

A.


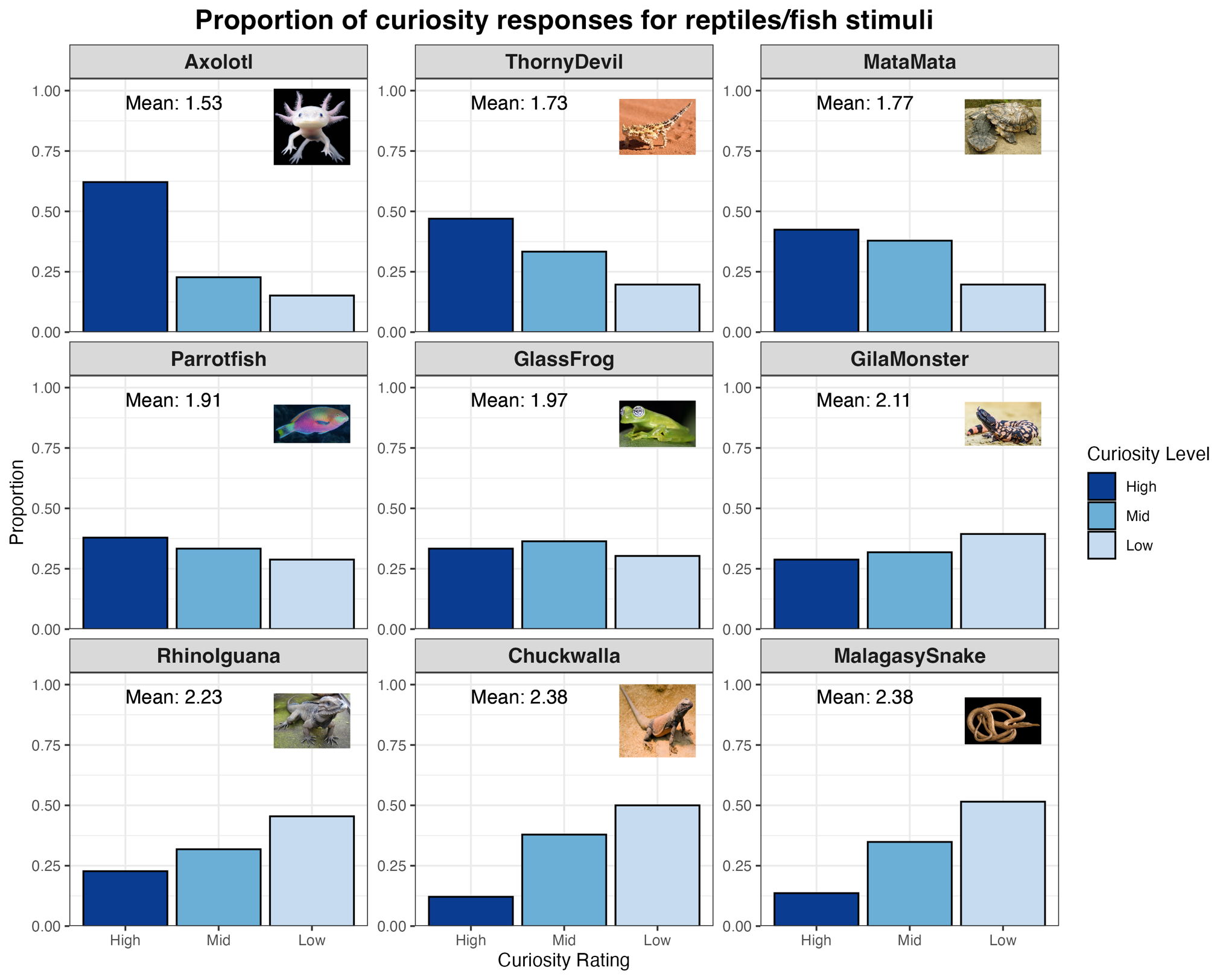


C.


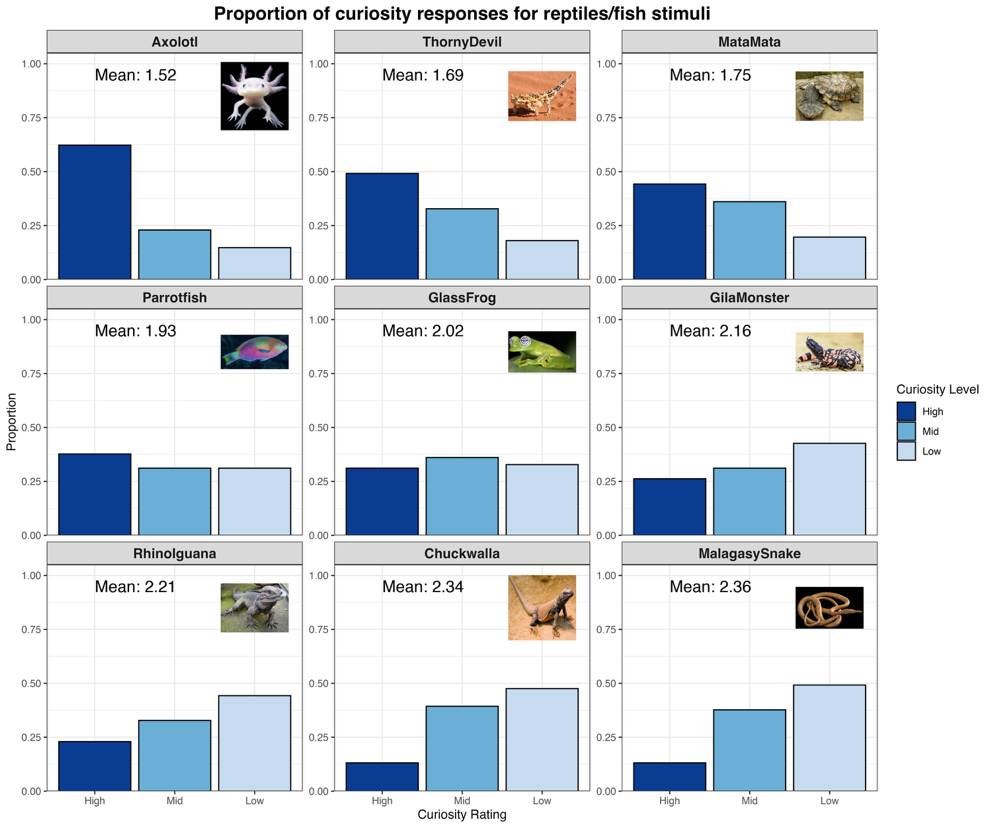


**Figure S2.** Proportion of high, mid, and low curiosity ratings for animal stimuli**. (A)** Birds, **(B)** mammals, and **(C)** reptiles/fish are shown.


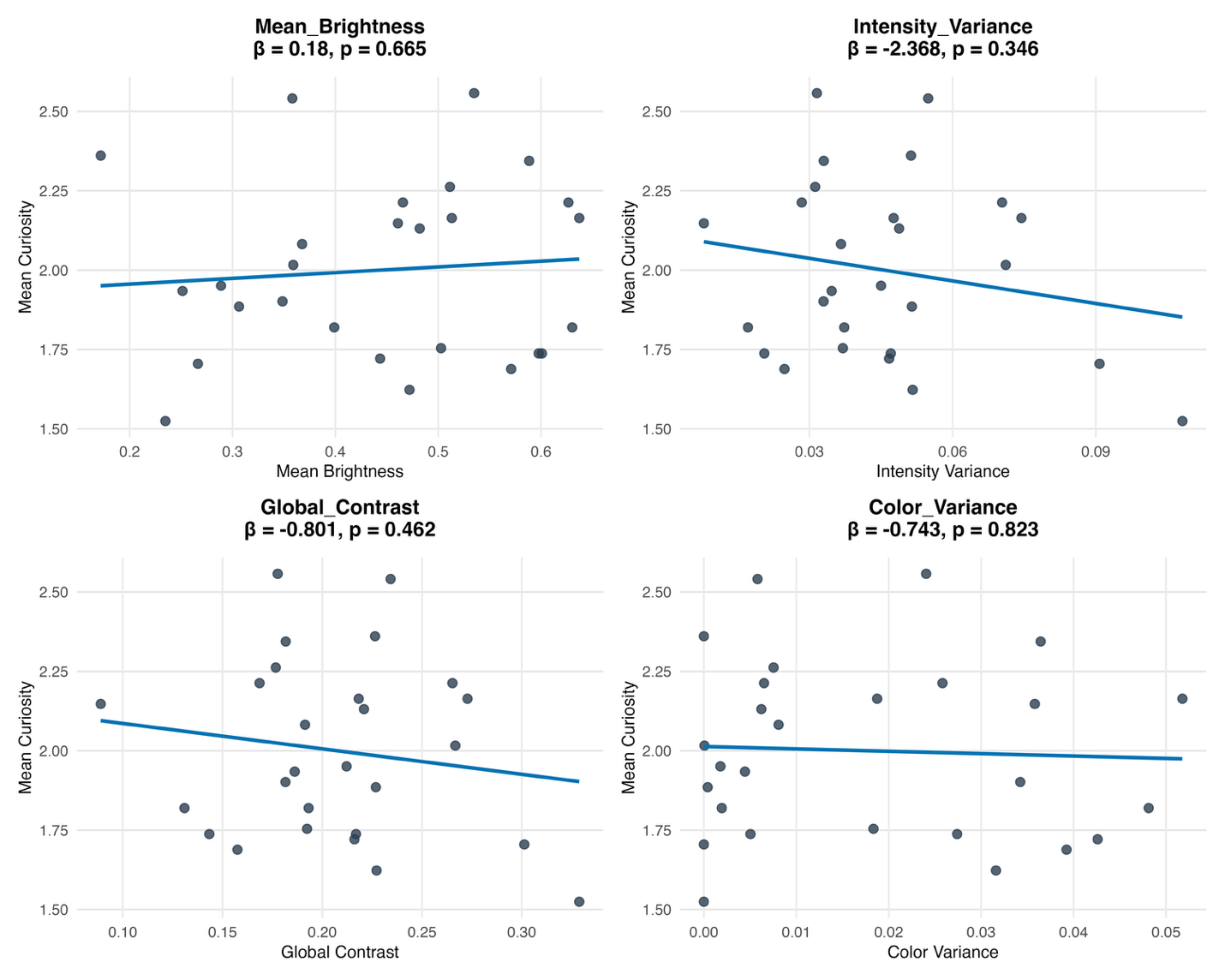


**Figure S3.** Mean curiosity ratings by perceptual properties of animal images. Mean brightness reflects the average grayscale intensity of image pixels (higher values indicate lighter grayscales). Intensity variance reflects the variance of grayscale intensity across image pixels (high values indicate dynamic light/dark areas). Global contrast reflects the standard deviation of grayscale intensities (higher values indicate more distinguishable image features). Color variance reflects the average variance in the red, green, and blue color channels (higher variance indicates more colorful complexity). All visual features were extracted using the magick package in R (Ooms, 2025; Pedersen, 2025).


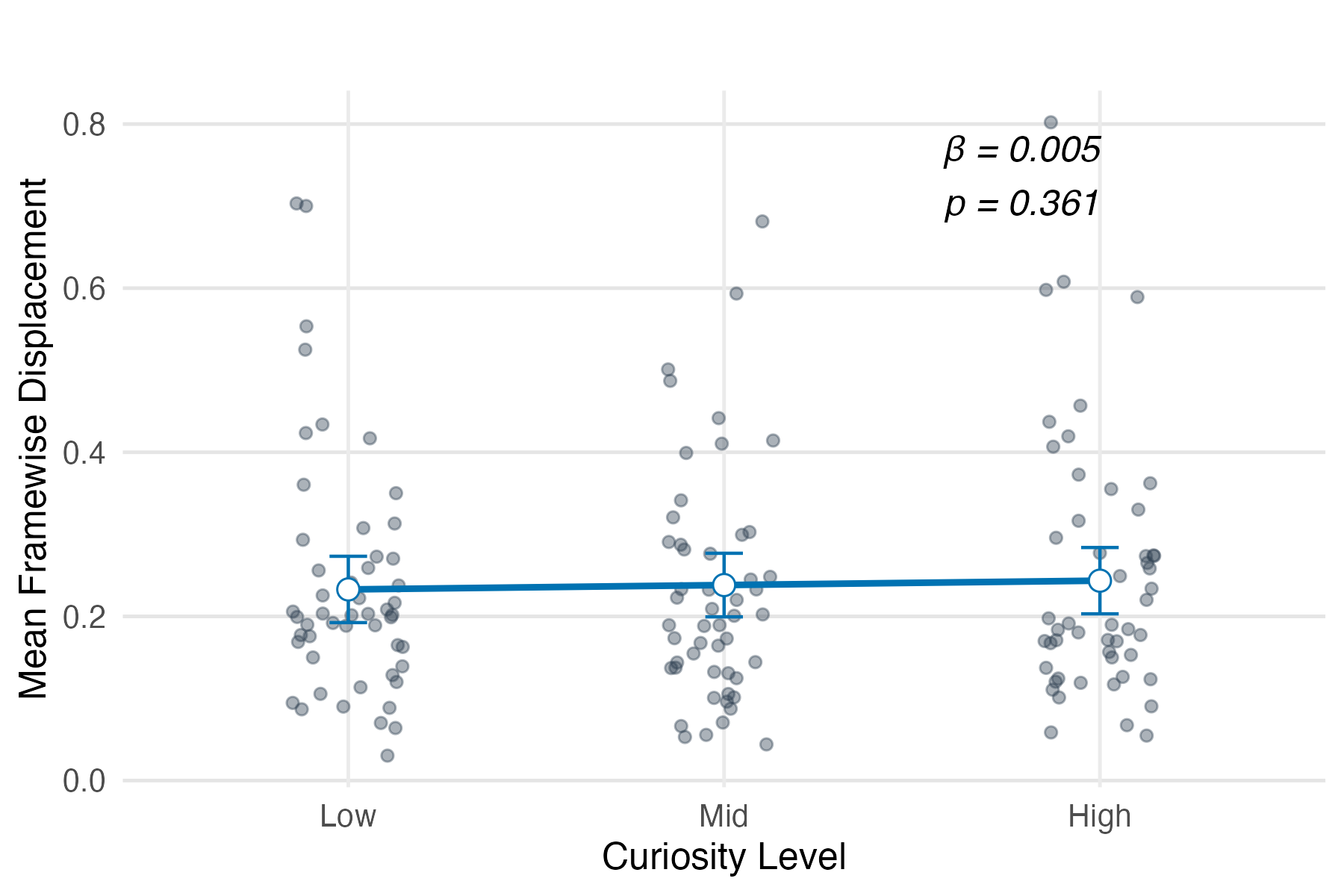


**Figure S4.** Children’s mean framewise displacement as a function of curiosity. Framewise displacement was recorded for each volume and averaged across trials for high-, mid-, and low- curiosity levels. Motion is shown for each child included in the fMRI sample (*n* = 51).

**
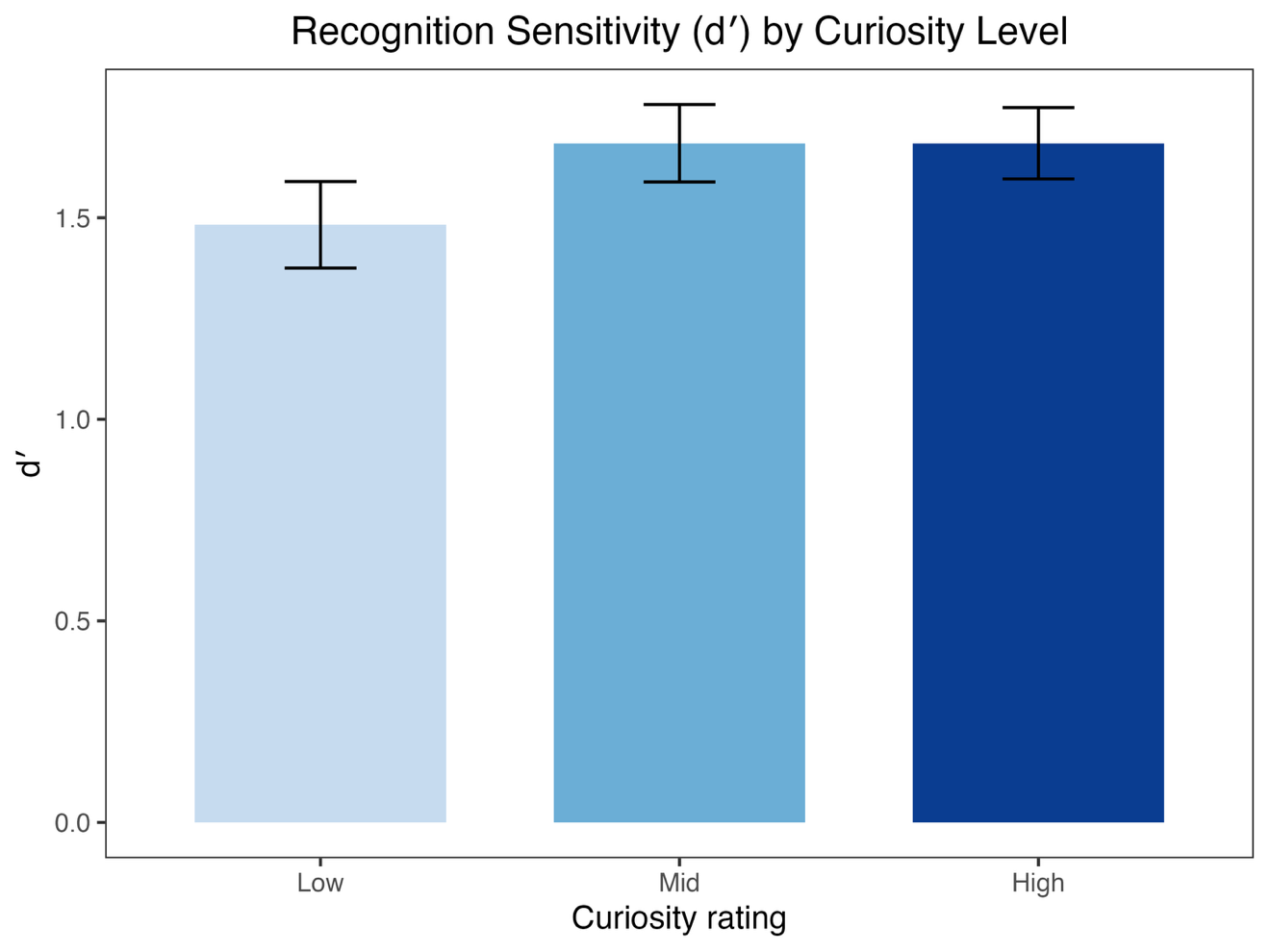
**

**Figure S5.** Mean recognition sensitivity (d’) for high-, mid-, and low-curiosity items. Error bars represent one standard error of the mean across participants. A linear mixed-effects model showed that higher curiosity ratings were associated with greater d’ (β = 0.10, SE = 0.04, *p* = 0.005).

**
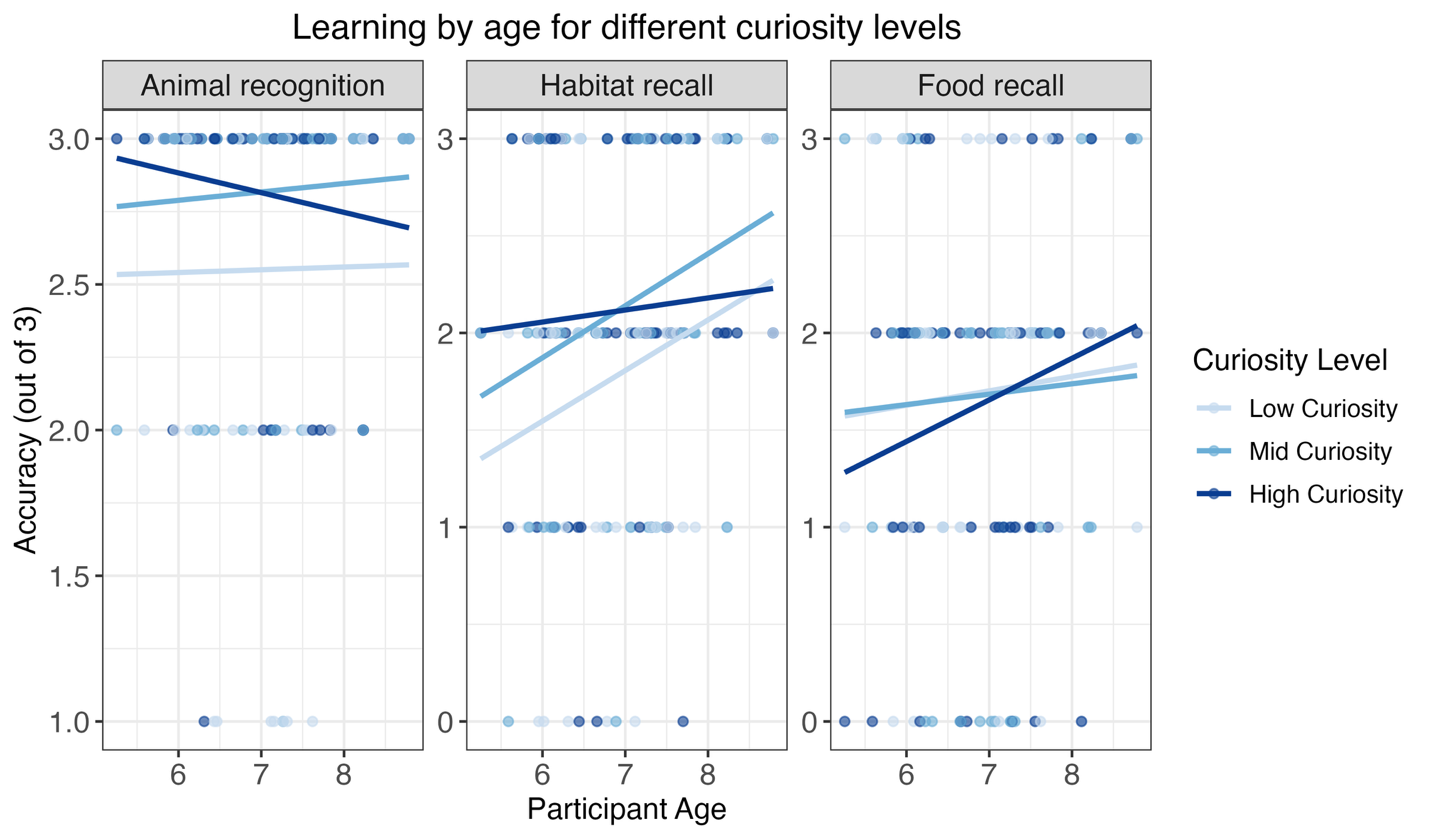
**

**Figure S6. Learning by age for different curiosity levels across task types.**
Children’s accuracy scores (out of 3) are shown by participant age for each trial type (recognition, habitat recall, and food recall). Linear regression lines illustrate trends for low (light blue), mid (medium blue), and high (dark blue) curiosity trials.


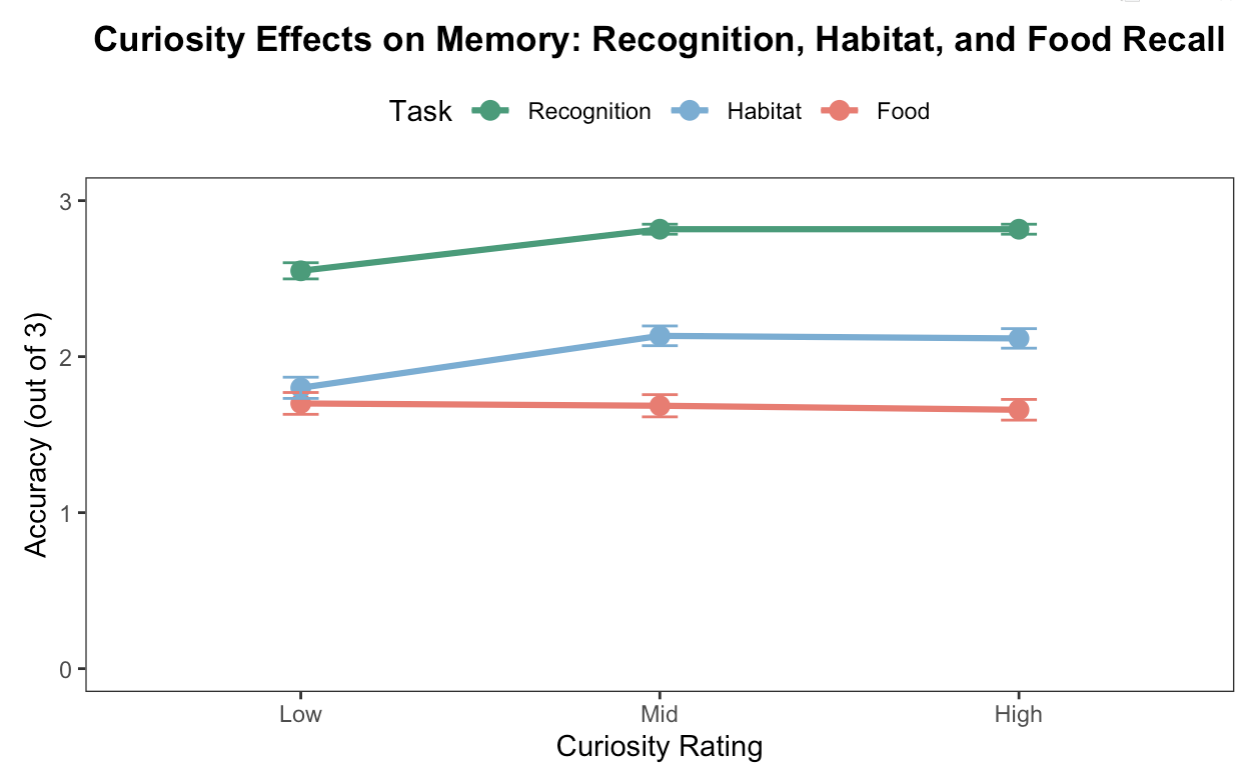


**Figure S7. Curiosity effects on memory performance across tasks.** Mean accuracy scores (out of 3) are shown for low-, mid-, and high-curiosity trials across the recognition, habitat, and food recall tasks. Error bars represent ±1 standard error of the mean. Accuracy was averaged across participants for each curiosity level and task type.

| **Table S2.** Behavioral mixed-effects model testing age, curiosity, and recall task type on memory performance | |  |
| --- | --- | --- |
|  |  |  |
| Age | 0.143* |  |
|  | (0.073) |  |
| Curiosity | 0.069* |  |
|  | (0.029) |  |
| Habitat recall task | 0.335*** |  |
|  | (0.048) |  |
|  |  |  |
| Constant | 0.545 |  |
|  | (0.513) |  |
| *Note:* The coefficient for the habitat recall task is estimated relative to the food recall task*.* Standard errors in parentheses.  ***p<.001; *p<.05 | |  |
|  |  |  |
|  |  |  |
|  |  |  |

| **Table S3.** Behavioral results excluding participants who performed below chance on each task | | | |
| --- | --- | --- | --- |
|  | Memory Task | | |
|  | A. Recognition | B. Habitat Recall | C. Food Recall |
| Age | -0.020 | 0.177* | 0.131 |
|  | (0.049) | (0.087) | (0.083) |
| Curiosity | 0.111* | 0.167* | -0.028 |
|  | (0.046) | (0.078) | (0.026) |
|  |  |  |  |
| Constant | 2.79*** | 0.671 | 0.893 |
|  | (0.348) | (0.616) | (0.586) |
| *Note:* Standard errors in parentheses. ***p<.001; *p<.05 | | | |


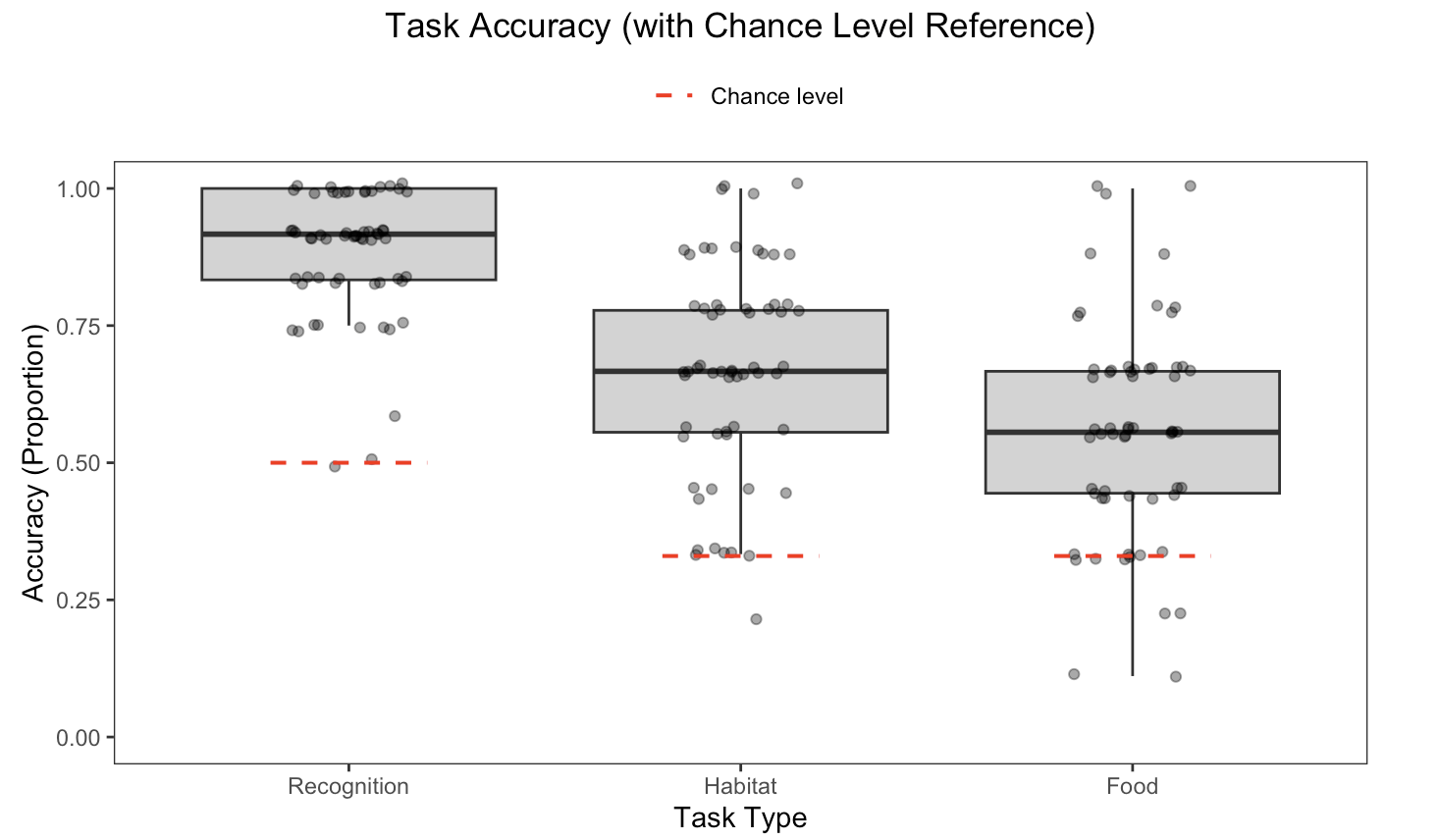


**Figure S8.** Participants’ accuracy across the recognition, habitat, and food recall tasks, shown as proportions correct. Each point represents one child’s mean accuracy for a given task, with boxplots summarizing group-level distributions. Dashed red lines indicate task-specific chance levels (50% for recognition; 33% for habitat and food recall).

**
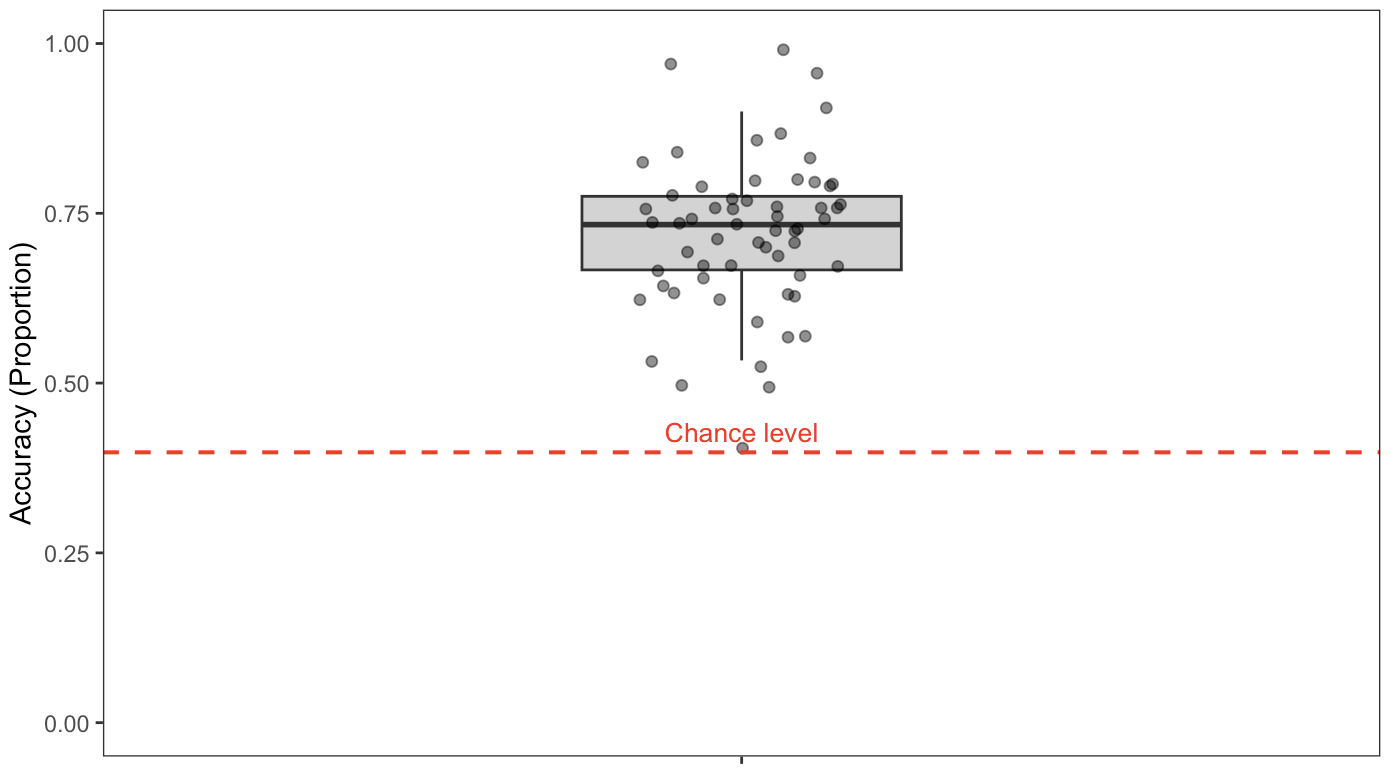
**

**Figure S9. Participants’ accuracy aggregated across recognition, habitat recall, and food recall tasks.** The dashed line indicates the overall chance-level accuracy (0.39), computed as the average expected accuracy across task types assuming random responses (0.50 for recognition and 0.33 for habitat and food tasks).

**
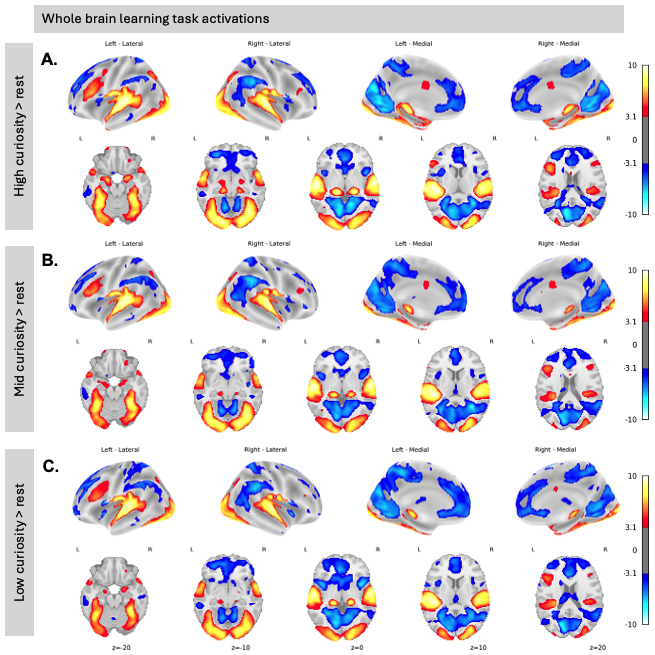
**

**Figure S10.** Neural activations during high-, mid-, and low-curiosity learning stimuli compared to rest. Analyses control for age and motion (mean framewise displacement). Maps show z-statistics corrected for false positive rates with a cluster threshold of 10 and z > 3.1.


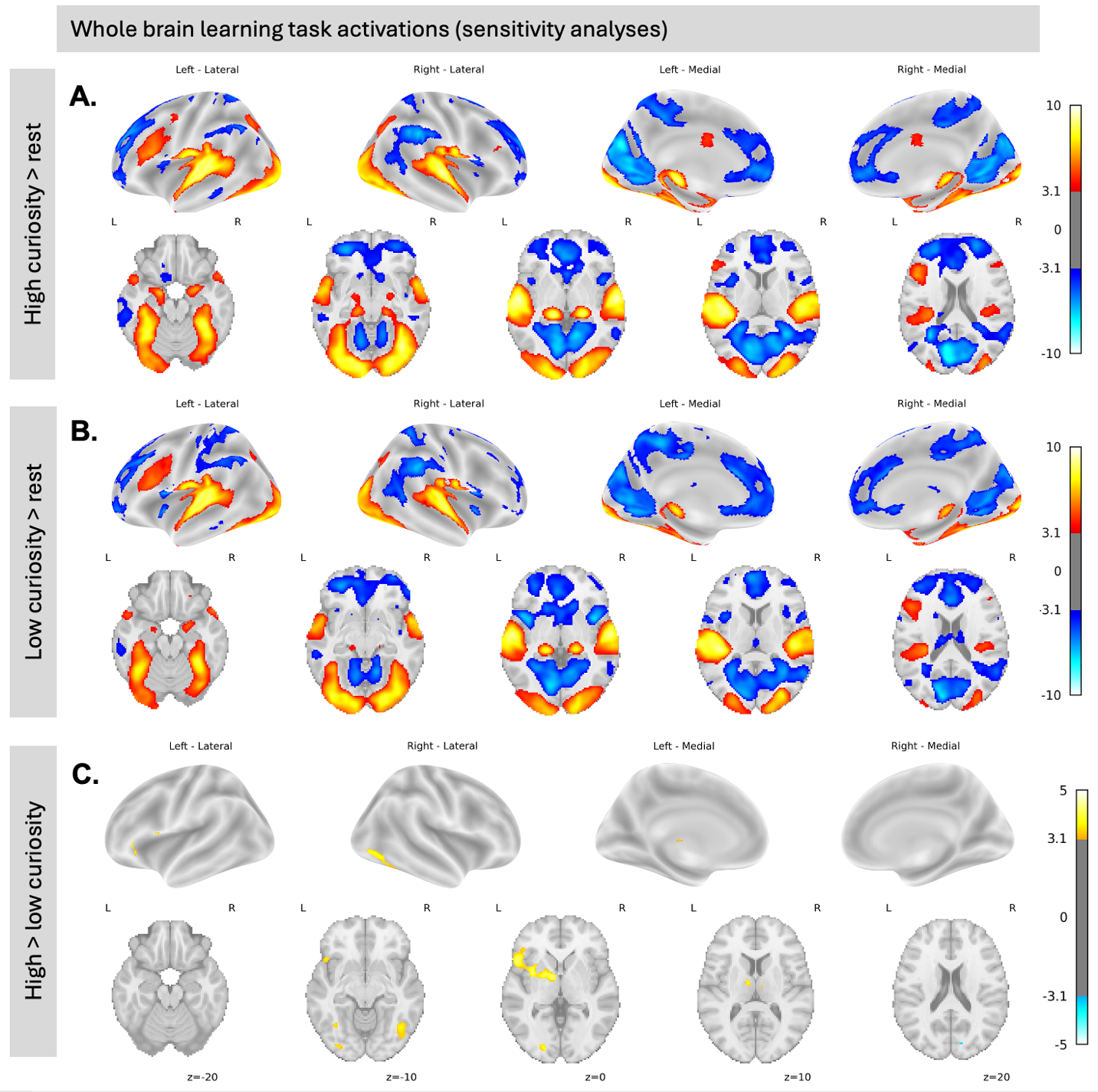


**Figure S11.** Sensitivity analyses showing neural activations during high- and low-curiosity learning stimuli compared to rest and high > low curiosity learning stimuli. Analyses exclude participants with > 0.5 mm mean framewise displacement in either task run and control for age and motion (mean framewise displacement). Maps show z-statistics corrected for false positive rates with a cluster threshold of 10 and z > 3.1.

**
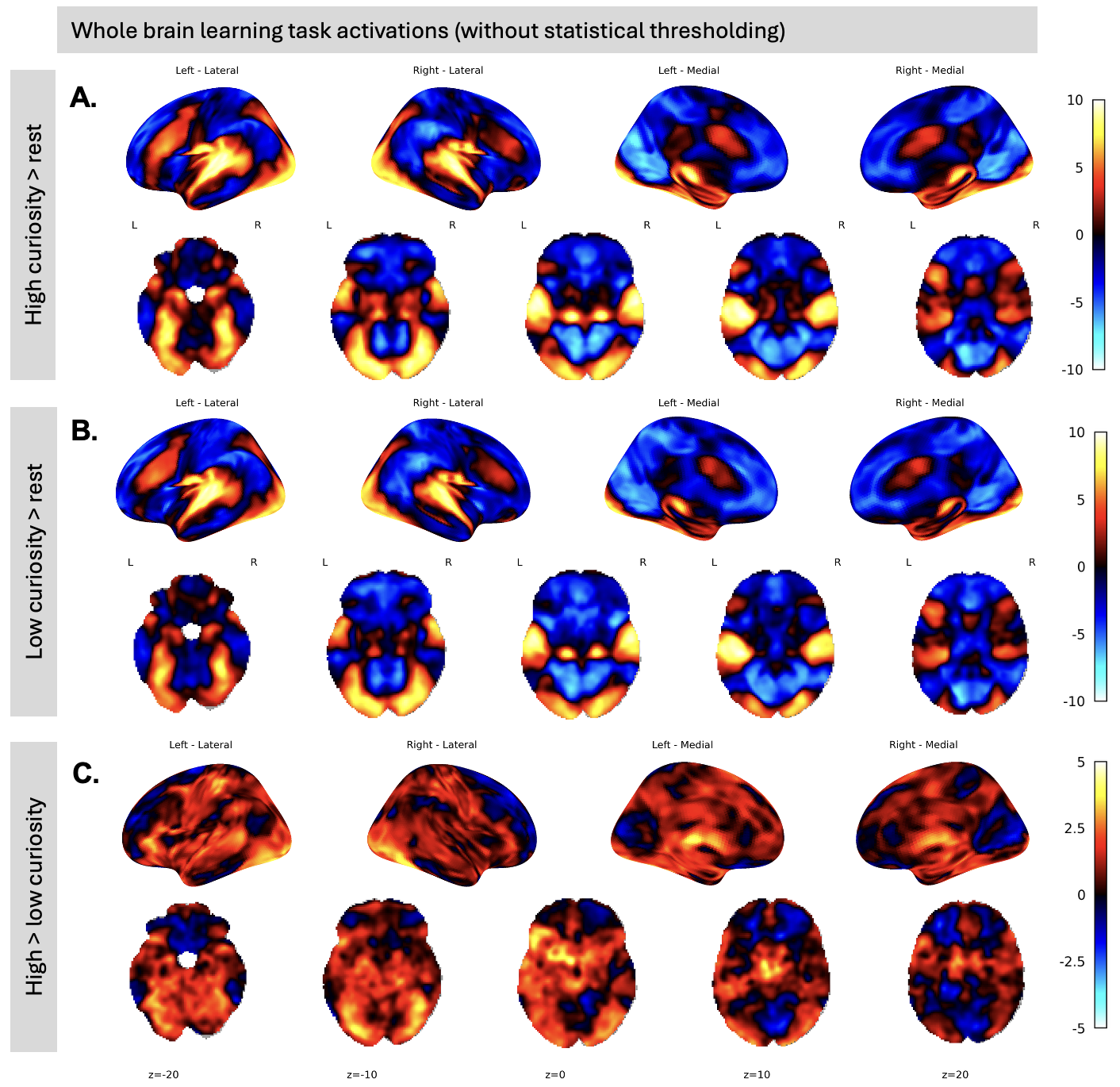
**

**Figure S12.** Neural activations during **A)** high- and **B)** low-curiosity learning stimuli compared to rest. Curiosity-related neural activations during **C)** high > low curiosity learning. Analyses control for age and motion (mean framewise displacement). Maps show z-statistics without statistical thresholding.

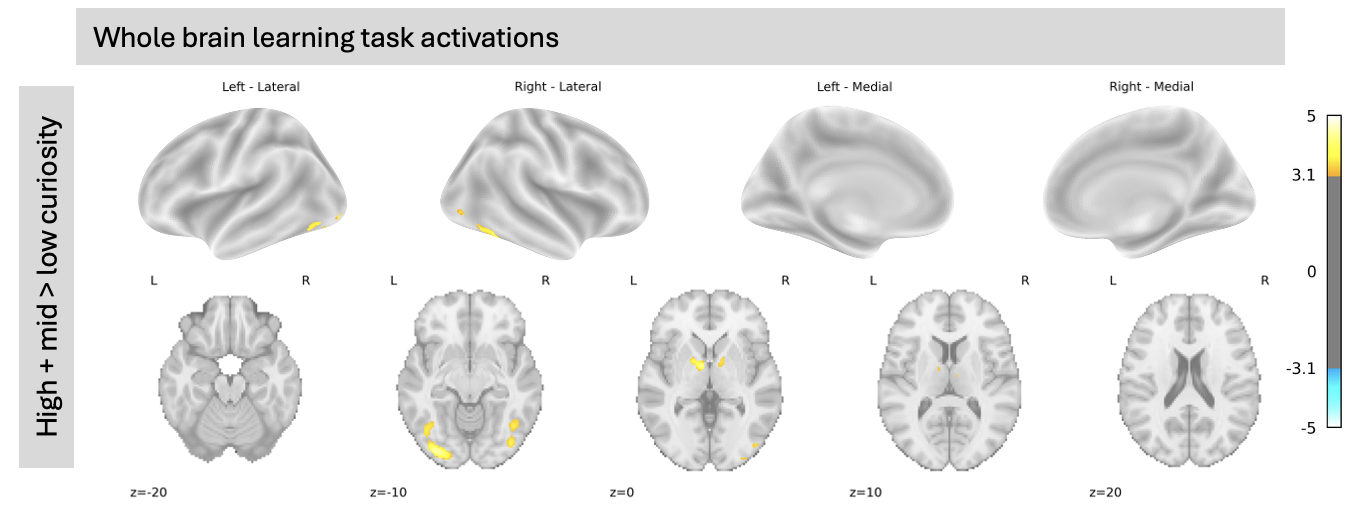


**Figure S13.** Neural activations during high- and mid- curiosity learning stimuli versus low-curiosity learning stimuli. Analyses control for age and motion (mean framewise displacement). Maps show z-statistics corrected for false positive rates with a cluster threshold of 10 and z > 3.1.

**
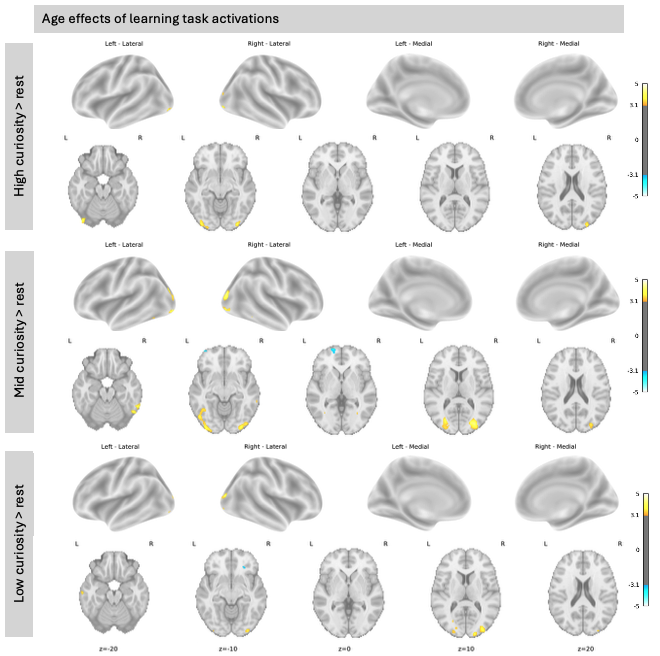
**

**Figure S14.** Age effects of neural activation during high-, mid-, and low-curiosity learning stimuli compared to rest. Analyses control for motion (mean framewise displacement). Maps show z-statistics corrected for false positive rates with a cluster threshold of 10 and z > 3.1. No age effects were observed in the high > low curiosity maps.

**
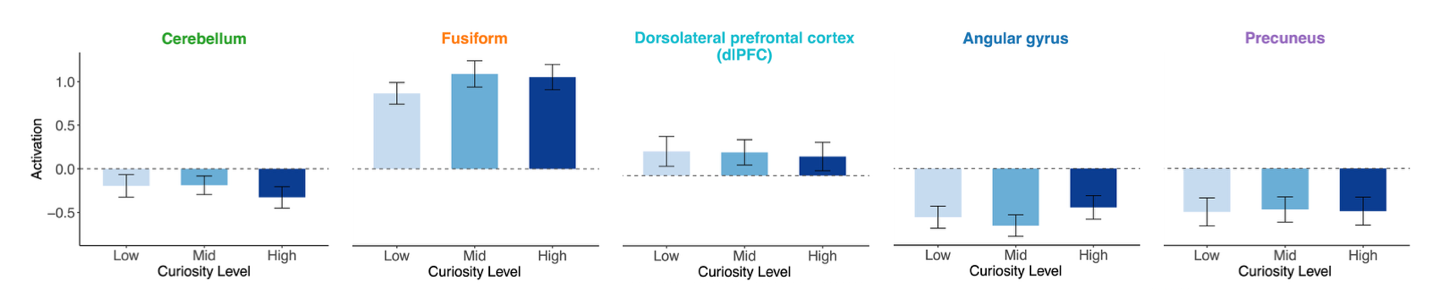
**

**Figure S15.** Parameter estimates of univariate activation are shown for high, mid, and low curiosity for each cluster identified by searchlight decoding to show whether these were task-positive or task-negative regions.

**Supplemental References**

Ooms J (2025). magick: Advanced Graphics and Image-Processing in R. R package version 2.8.6, <https://github.com/ropensci/magick>.

Pedersen T (2025). *patchwork: The Composer of Plots*. R package version 1.3.0.9000, https://github.com/thomasp85/patchwork, [https://patchwork.data-imaginist.com](https://patchwork.data-imaginist.com/).
